# Membrane-Enriched Proteomics Link Ribosome Accumulation and Proteome Reprogramming With Cold Acclimation in Barley Root Meristems

**DOI:** 10.3389/fpls.2021.656683

**Published:** 2021-04-30

**Authors:** Federico Martinez-Seidel, Pipob Suwanchaikasem, Shuai Nie, Michael G. Leeming, Alexandre Augusto Pereira Firmino, Nicholas A. Williamson, Joachim Kopka, Ute Roessner, Berin A. Boughton

**Affiliations:** ^1^School of BioSciences, The University of Melbourne, Parkville, VIC, Australia; ^2^Willmitzer Department, Max-Planck-Institute of Molecular Plant Physiology, Potsdam-Golm, Germany; ^3^Bio21 Institute of Molecular Science and Biotechnology, The University of Melbourne, Parkville, VIC, Australia; ^4^School of Chemistry, The University of Melbourne, Parkville, VIC, Australia; ^5^Department of Biochemistry and Molecular Biology, The University of Melbourne, Parkville, VIC, Australia; ^6^Australian National Phenome Centre, Murdoch University, Murdoch, WA, Australia

**Keywords:** *Hordeum vulgare*, cv Keel, abiotic stress, translation, ribosomal protein paralog, fungal priming elicitors, chitin, chitosan

## Abstract

Due to their sessile nature, plants rely on root systems to mediate many biotic and abiotic cues. To overcome these challenges, the root proteome is shaped to specific responses. Proteome-wide reprogramming events are magnified in meristems due to their active protein production. Using meristems as a test system, here, we study the major rewiring that plants undergo during cold acclimation. We performed tandem mass tag-based bottom-up quantitative proteomics of two consecutive segments of barley seminal root apexes subjected to suboptimal temperatures. After comparing changes in total and ribosomal protein (RP) fraction-enriched contents with shifts in individual protein abundances, we report ribosome accumulation accompanied by an intricate translational reprogramming in the distal apex zone. Reprogramming ranges from increases in ribosome biogenesis to protein folding factors and suggests roles for cold-specific RP paralogs. Ribosome biogenesis is the largest cellular investment; thus, the vast accumulation of ribosomes and specific translation-related proteins during cold acclimation could imply a divergent ribosomal population that would lead to a proteome shift across the root. Consequently, beyond the translational reprogramming, we report a proteome rewiring. First, triggered protein accumulation includes spliceosome activity in the root tip and a ubiquitous upregulation of glutathione production and *S*-glutathionylation (S-GSH) assemblage machineries in both root zones. Second, triggered protein depletion includes intrinsically enriched proteins in the tip-adjacent zone, which comprise the plant immune system. In summary, ribosome and translation-related protein accumulation happens concomitantly to a proteome reprogramming in barley root meristems during cold acclimation. The cold-accumulated proteome is functionally implicated in feedbacking transcript to protein translation at both ends and could guide cold acclimation.

## Introduction

Cereals face two kinds of low-temperature challenges. The first is vernalization (Chouard, 1960; Von Zitzewitz et al., 2005; Deng et al., 2015), which is bound to seasonal climate and acts as a cue to induce the transition from vegetative to reproductive development. The second is cold acclimation (Thomashow, 1999; Miura and Furumoto, 2013), which occurs in response to sudden cold stimuli and requires phenotypic plasticity to respond. Plants acclimate to low temperatures using a systemic response (Hincha and Zuther, 2020) that ranges from hormonal, auxin-dependent signaling (Shibasaki et al., 2009; Rahman, 2013), through primary metabolism (Kaplan et al., 2004; Zuther et al., 2019; Erban et al., 2020), and to membrane-lipidome compositional changes (Uemura et al., 1995; Cheong et al., 2019, 2020). Typical cold responses have been divided according to the response time after the initial stimulus (Seki et al., 2002) and involve proteins that function as the principal modulators. A well-known example of an immediate cold response is the C−repeat binding factor (CBF) regulatory pathway (Jaglo-Ottosen et al., 1998; Thomashow, 1999; Fowler and Thomashow, 2002) that operates a transcriptional regulon both in dicots (Park et al., 2015) and in monocots (Novák et al., 2017). On the other hand, long-term responses are less well understood and could partially rely on more permanent changes, for instance, by including altered protein translation or ribosomes as a main hub of the cold response or memory (Beine Golovchuk et al., 2018; Garcia-Molina et al., 2020; Cheong et al., 2021).

At the physiological level, changes occurring due to cold acclimation are coupled to molecular mechanisms in the roots from the model dicot *Arabidopsis thaliana* (Ashraf and Rahman, 2019). *Arabidopsis* roots respond to 4°C by reducing root growth, specifically mitotic division but not cell elongation, which significantly decreases the size of the meristem. A partial growth arrest occurs because of altered trafficking of auxin efflux carriers. The resulting imbalance in the intracellular auxin concentrations is caused by inhibition of the ARF guanine-nucleotide exchange factor GNOM (Ashraf and Rahman, 2019), which functions as an ADP ribosylation/GTP exchange factor (ARF-GEF; Steinmann et al., 1999). Reduced GNOM abundances delay papillae formation, which are localized cell-wall appositions implicated in plant innate immunity, e.g., against barley powdery mildew fungus (Nielsen et al., 2012). Consequently, the inhibition of GNOM during cold stress could render plants more susceptible to fungal pathogens. Contrary to expectations, cold stress seems to improve immunity against fungi in cereals (Płazek et al., 2003; Kuwabara and Imai, 2009), where linked mechanisms are even less clear, indicating some level of unknown cellular remodeling.

Monocots, which include cultivated cereal crops, and dicots share convergent evolution and common ancestors. Barley root systems (*Hordeum vulgare* L.), for instance, have unique and conserved features (Kirschner et al., 2017) that outline root evolution. Thus, interchangeable insights from shared molecular mechanisms routinely provide design rationale for crop science improvement. Expanding upon our understandings of root molecular physiology will further detail unique and conserved mechanisms across plant lineages, where the current gaps extend to root spatial proteome composition, biological steady states, and plasticity. The landscape of root cells is intrinsically heterogeneous, with cells coexisting in multiple biological steady states (Dinneny and Benfey, 2008), while meristems establish the primary root and machinery to import and utilize nutrients (Nelson et al., 2014), and differentiated root tissue matures and engages in metabolism. These transient developmental states serve as a basal level to compare the proteome of plants challenged with biotic or abiotic stress cues. Moreover, by carefully studying specific developmental stages, a quasi-steady state rate of turnover for subsets of proteins might be assumed (Huffaker and Peterson, 1974), allowing clear distinctions between unperturbed and challenged plant proteomes. Spatially, compositional changes in the plant proteome can be magnified in root zones with high protein production rates such as meristem apexes and cell division zones (Clowes, 1958; Verbelen et al., 2006).

Cold responses can be traced back to proteins and proteome shifts (Janmohammadi et al., 2015) affecting root molecular physiology. Studying the *in planta* responsiveness of the root proteome to cold presents its own difficulties. For instance, hydroponic systems imply adding exogenous sugar to the growth media and/or triggering roots with undesired light stimuli. Additionally, harvesting of whole root systems precludes distinguishing rapid proteome changes because newly synthesized and preexisting proteins are pooled together. The impact of these limitations can be minimized using prolonged stratification to study cold acclimation in root tips. Seed stratification, an early cold cue applied to imbibed seeds during germination, is comparable in magnitude with low temperature plant acclimation. Stratification enhances and synchronizes seed germination through the action of reactive oxygen species (ROS) oxidizing biomolecules and acting in concert with phytohormones (Su et al., 2016; Bailly, 2019). However, we argue that extended and maintained stratification triggers acclimation. First, the cold stimulus induces upregulation of the protein post-translational modification (PTM), specifically the *S*-glutathionylation machinery that could act proteome-wide as will be described in this study. Low temperature induction of both thiol-modifying and antioxidative machineries has been reported in plants (Koehler et al., 2012; Golemiec and Gołȩbiowska-Pikania, 2015). Glutathione is added to newly formed proteins via cysteine residues where they act as ROS scavengers (Diaz-Vivancos et al., 2015). Second, typical cold acclimation triggers a transcriptome-wide reprogramming characterized by a rapid increase of transcript splicing variants (Calixto et al., 2018). Spliceosome activity co-occurs with a vast translational reprogramming in root apexes (this study). Thus, the mentioned observations along with accumulation of cold markers in our datasets ratify stratified barley root tips as a legitimate study system for plant proteome cold acclimation. Additionally, these observations warrant exploration as to whether they are linked. This hypothesis stands to reason and could involve cold-specialized ribosomes in plants (Martinez-Seidel et al., 2020) that may select alternatively spliced transcripts for translation while generating feedback on the PTM machinery.

Here, we uncover previously hidden aspects of cold proteome-wide responses that are concomitant to a highly committed ribosome accumulation. We provide an overview of the changes in the apical and adjoined more distal seminal root segments. The biological responses are clear and specific for each root zone but share commonalities. An observed translational reprogramming event near the root meristem, where the cold triggered ribosome accumulation happened, led us to analyze homology between both *Arabidopsis* ribosome-associated proteins (RAPs) and ribosomal protein (RP) families and barley homologs. This information was previously not available and allowed us to test for and compare cold-specific RAP and RP paralogs. Finally, we outline cold-triggered shifts of the proteome where specific molecular functions were enhanced or diminished. As an example, we discuss seedling priming for biotic stress, which could be interpreted as both enhanced and diminished by cold in our dataset. Thus, in order to understand how cold-acquired resistance arises, we treated the plants with conventional fungal priming elicitors, chitin and chitosan, as they would be treated in the field. This allowed us to understand that the priming elicitors do not trigger significant proteome shifts that are shared with cold derived responses and thus speculate on how cold-acquired resistance might stem from different cellular processes in barley roots as compared with elicitor-induced resistance.

## Materials and Methods

### Growth Conditions

*Hordeum vulgare* cv Keel seeds were sourced from The University of Melbourne from previous studies (Gupta et al., 2019). Parental plants were grown under optimized conditions for seed production. Seeds were surfaced-sterilized in 70% (1 min) ethanol and 1% bleach (10 min) and washed. Imbibition lasted 12 h. Seeds were transferred to Petri dishes containing a filter paper and 3 cm^3^ Hoagland medium. For priming, 1% chitin or chitosan was mixed (Lowe et al., 2012; Cretoiu et al., 2013). Dishes were left in the dark at 25°C/18°C (16 h/8 h), inside a phytotron growth chamber (Weiss Technik, Germany) for 48 h of germination. Then half of the dishes were shifted to 4°C. Seeds were germinated for a total of 7 days. The treatments were as follows: control (7 days at 25°C/18°C) with or without 1% chitin or chitosan priming compounds, and cold (2 days control germination and 5 days at 4°C). Roots were cut and instantly frozen; ∼1.5-cm seminal root (Supplementary Figure 1) segment pools were ground in liquid nitrogen, separated in ∼40-mg aliquots, and stored at −80°C.

### Proteomics Profiling

#### Protein Extraction

Aliquots were solubilized in 200 mm^3^ of ribosome extraction buffer (REB), incubated on ice for 20 min, and centrifuged at 17,900 × *g* for 10 min at 4°C. Supernatant was loaded into a QIAshredder (Qiagen) and centrifuged full-speed for 1 min; 400 mm^3^ of 6 M guanidine-HCl was added to the flow through, followed by 4 mm^3^ of neat trifluoroacetic acid (TFA). Centrifugation was repeated to precipitate out RNA. The final supernatant was reserved. The initial pellet was solubilized in 600 mm^3^ of lysis buffer (LB), vortexed, incubated at 95°C for 3 min, and sonicated for 5 min. Centrifugation was carried out as before, and supernatant was collected. Both supernatants were mixed with -20°C precooled 10% trichloroacetic acid (TCA) dissolved in acetone and freshly supplemented with 1× protease inhibitor cocktail (Sigma, P9599) for 16 h. The solution was centrifuged at 17,900 × *g* for 10 min. Finally, the protein pellet was washed twice with -20°C acetone and air-dried.

Ribosome extraction buffer was prepared as previously described (Firmino et al., 2020) without sodium deoxycholate (DOC).

Lysis buffer was 4% sodium dodecyl sulfate (SDS); 100 mM of Tris/HCl, pH 7.4; 1× protease inhibitor cocktail (Sigma, P9599).

#### Ribosomal Protein Content

Modified REB was used in order to extract ribosomes from a total of six biological replicates (*n* = 6). Briefly, the detergent mix was replaced by octyl beta-D-glucopyranoside (≥98% Sigma-Aldrich, O8001) concentrated 0.01 M above the critical micelle concentration of 0.025 M in order to hold micelles after cell lysis. Diluted extracts (3.5×) derived from 200 mg of fresh weight (FW) were centrifuged at 330,000 × *g* for 4.5 h on a 70.1Ti rotor (Type 70.1 Ti Rotor, Beckman Coulter, United States). Extracts were layered inside thick-walled polycarbonate tubes with three-piece caps (10.4 cm^3^, polycarbonate bottle with cap assembly, 16 mm × 76 mm—6Pk, 355603, Beckman Coulter, United States) on top of 2.5 cm^3^ of 30% sucrose cushion (SC) solution.

Sucrose cushion was 0.4 M of Tris, pH 9.0, 0.2 M of KCl, 0.005 M of EGTA, pH 8.0, 0.035 M of MgCl_2_ × 6H_2_O, and sucrose (Molecular Biology Grade, 573113, Sigma-Aldrich, Australia). Additions before extraction were 0.15 mM of chloramphenicol, 0.18 mM of cycloheximide, and 5 mM of DTT.

Pure *Escherichia coli* ribosomes (P0763S, NEB, Australia) were ultracentrifuged following the same procedure as a control of the ribosomal particles passing the cushion. After ultracentrifugation, pellets enriched in ribosomes were diluted in 6 M of GuHCl, acidified to 1% TFA, and centrifuged at max speed in a benchtop microcentrifuge. The supernatant was recovered, and protein content was evaluated using the bicinchoninic acid (BCA) kit (Thermo Scientific, United States) assay as detailed in the following section.

#### Protein Digestion

Protein pellets were resuspended using 8 M of urea in 50 mM of triethylammonium bicarbonate (TEAB), pH 8.5, sonicated for 20 min at 37°C, and centrifuged at 20,627 × *g* for 2 min. Protein contents were measured using the BCA assay (see section “Induced Changes of the Root Proteome”). Protein of 1 mg/cm^3^ was reduced with tris(2-carboxyethyl)phosphineı (TCEP; 10 mM of final concentration) at 37°C for 45 min and alkylated with iodoacetamide (IAA; 55 mM of final concentration) at 37°C for 45 min in the dark. Solution was diluted with 25 mM of TEAB at pH 8.5 to 1 M of urea. Trypsin (Pierce Trypsin Protease, mass spectrometry grade, Thermo Fisher Scientific) in 25 mM of TEAB was added to the samples (1:40) and shaken overnight at 37°C. TFA was added to 1% final volume. Peptides were loaded in Oasis solid-phase extraction (SPE) cartridges (Waters Co., United States), washed with 0.1% TFA, and eluted out using 80% acetonitrile (ACN) with 0.1% TFA. Peptides were dried in a vacuum concentrator (Savant ISS110, Thermo Fisher Scientific) and a freeze-dryer (Alpha 3-4 LSCbasic, Christ).

#### Tandem Mass Tag Labeling

Tandem mass tag (TMT) labeling was done as previously reported (Zecha et al., 2019) with minor modifications. Ten micrograms of peptides was labeled with 4 mm^3^ of 6-plex TMT labeling reagent (0.8 mg of TMT in 41 mm^3^ of ACN). Labeling reaction was incubated for 1 h at 25°C and 400 rpm in a benchtop-shaking incubator. One cubic millimeter of 5% hydroxylamine was added and incubated for 15 min as before. Finally, 10 mm^3^ of labeled peptides from each sample was mixed and cleaned using the SPE cartridge procedure. Cleaned peptides were resuspended in MS loading buffer (2% ACN, 0.05% TFA) and loaded into a nano-electrospray ionization–liquid chromatography–tandem MS (nano-ESI-LC-MS/MS).

#### LC-MS/MS Analyses

The nano-LC system, Ultimate 3000 RSLC (Thermo Fisher Scientific), was equipped with an Acclaim PepMap nano-trap column (C18, 100 Å, 75 μm × 2 cm, Thermo Fisher Scientific) and an Acclaim PepMap RSLC analytical column (C18, 100 Å, 75 μm × 50 cm, Thermo Fisher Scientific) maintained at a temperature of 50°C. For each LC-MS/MS experiment, 1 μg of peptides was loaded onto the enrichment (trap) column at an isocratic flow of 5 mm^3^/min of 3% ACN containing 0.05% TFA for 6 min before the enrichment column was switched in-line with the analytical column. The eluents used for the LC were water with 0.1% v/v formic acid and 5% v/v dimethyl sulfoxide (DMSO) for solvent A, and ACN with 0.1% v/v formic acid and 5% DMSO for solvent B. The gradient used (300 nl/min) was from 3% B to 23% B for 144 min, 23% B to 45% B in 10 min, and 45% B to 80% B in 10 min and maintained at 80% B for the final 5 min before dropping to 3% B in 1 min and equilibration for 9 min at 3% B prior to the next analysis. The MS experiments were performed using a nano-ESI source at positive mode and Orbitrap Eclipse mass spectrometer (Thermo Fisher Scientific, San Jose, CA, United States). The spray voltages, capillary temperature, and S-lens RF level were set to 1.9 kV, 275°C, and 30%. The MS data were acquired with a 3-s cycle time for one full-scan MS spectra and as many data-dependent higher-energy collisional dissociation (HCD)-MS/MS spectra as possible. Full-scan MS spectra had a *m/z* of 375–1,500, a resolution of 120,000 at *m/z* 200, an automatic gain control (AGC) target value of 4e^5^, and a maximum ion trapping time of 50 ms. The data-dependent HCD-MS/MS of precursor ions (charge states from 2 to 7) was performed using an *m/z* isolation window of 1.6, a first mass at *m/z* of 100, an AGC target value of 5e^4^, a normalized collision energy (NCE) of 30%, a resolution of 15,000 at *m/z* 200, and a maximum ion trapping time of 22 ms. Dynamic exclusion was used for 30 s.

### Data Acquisition and Interpretation

#### Barley Proteome

The recovered proteome from different extraction methods was evaluated from a total of three biological replicates (*n* = 3, Supplementary Figure 2). *H. vulgare* gene IDs and FASTA sequences (Supplementary Table 1) were obtained by aligning Swiss-Prot entries (351 reviewed proteins downloaded on 21. March 2020, “uniprot-hordeum + vulgare-filtered-reviewed_yes + AND + organism__Hordeum + vulgar–.fasta”) with high-confidence proteogenomics annotations.^1^ The same annotations were then used to evaluate proteome shifts occurring due to experimental conditions from a total of five biological replicates (*n* = 5, Supplementary Figure 3). Gene ontology (GO) terms were obtained by intersecting *H. vulgare* GOs from the amiGO database (Carbon et al., 2009; Mi et al., 2019) and the GOexpress (Rue-Albrecht et al., 2016) R package (Supplementary Figure 4 and Supplementary Table 1). Ontology assignments were performed using GO enrichment (the GO resource) (Carbon et al., 2009; Mi et al., 2019). Outputs were hierarchically sorted and interpreted as a group. The false discovery rate (FDR)-corrected Fisher exact test was applied. Lists of GOs were inputted into REVIGO (Supek et al., 2011) to remove redundant GOs and build semantic similarity-based scatterplots Supplementary Table 2.

#### Homology Alignments

A function to align two FASTA files was written. The usage is detailed in a GitHub repository.^2^ The alignment is a dependency of the pairwiseAlignment R function (Durbin et al., 1998; Haubold and Wiehe, 2006; Malde, 2008).^3^ pairwiseAlignment solves the global (Needleman and Wunsch, 1970), local (Smith and Waterman, 1981), or (ends-free) overlap pairwise sequence alignment problems. BLAST (Altschul et al., 1990), in comparison, is a development of the Smith–Waterman algorithm. Accuracy is significantly better in the Smith and Waterman as compared with BLAST (Pearson, 1995; Shpaer et al., 1996). However, the efficiency is lower in the Smith and Waterman global alignment. All alignments were performed with the default settings of the pairwiseAlignment function, i.e., global, which is equivalent to the online version of BLAST that has an interface for global (Needleman–Wunsch) alignment.^4^ Alignment scores were treated as a relative scale where the largest numbers represent the best alignments and comparisons can be then drawn for lower scores. Alignment scores >10 were selected as acceptable for potential homology based on the examples provided in the package documentation.

#### Protein Contents

Protein contents (*n* = 5) were fitted in a linear regression of bovine serum albumin (BSA) (Supplementary Table 3). Values were transformed into μg/mg of FW by normalizing to initial weight. The Shapiro–Wilk test and the Brown–Forsythe Levene-type test (bootstrapped when *n* > 10) were done to test normality and homoscedasticity. Kurtosis and square of skewness defined the distribution shapes of variables (Supplementary Figure 5). Variable distributions were plotted in a scatterplot along with exemplary distributions as detailed in RandoDiStats.^5^ Based on the known distributions, generalized linear models (GLMs) with an appropriate link function were used to test mean differences.

#### Shotgun Proteomics

##### Pre-processing

Proteomics data have been deposited to the ProteomeXchange Consortium (Deutsch et al., 2020) via the PRIDE (Perez-Riverol et al., 2019) partner repository with the dataset identifier PXD021731. RAW chromatograms were processed with MaxQuant, version 1.6.10.43 (Cox and Mann, 2008). TMT data correction (Thompson et al., 2003) was performed using the relative enrichment percentages of the tags. Isotope purity-corrected reporter ion intensities were obtained for each isobaric labeling channel summed over all MS/MS spectra matching to each protein group from the MaxQuant search. Search parameters include the following: (1) fixed—carbamidomethyl (C) and variable—oxidation (M), acetyl (protein N-term) modifications. (2) Allowed missed cleavages = two. Everything else was set as default. The TMT-corrected intensity matrix was imported into Perseus (Tyanova et al., 2016). Intensities were log_2_ transformed and normalized to pooled samples (analyzed together with each 6-plex TMT sample), enabling relative quantitation between root zones. Reverse search rows were used to adjust the annotations using the FDR (1%), complemented by acceptance of razor + unique peptides (UP) with a collective value of two or more. A multitude of volcano plots, a.k.a. a Hawaii plot, was built using Perseus (Tyanova et al., 2016). The log_2_ ratio between samples and controls was plotted in the *x*-axis against -log_10_(*P* adjusted values) in the *y*-axis (Supplementary Figure 3).

##### Statistics

Class discovery was applied on a non-scaled log_2_-transformed matrix (Supplementary Table 4, columns B–AN); proteins were clustered by covariance, enabling to find correlated proteins with large absolute changes, i.e., highly abundant in roots. Values in cells are proportions relative to pooled samples. Hierarchical cluster analysis (HCA) was performed using Pearson correlation as distance and average clustering as building method. Clustering was bootstrapped 10,000 and 1,000 times for treatments and proteins, respectively. “Approximately unbiased” (AU) *P* values were calculated using the R package pvclust (Suzuki and Shimodaira, 2006). Heatmaps were built using the R package ComplexHeatmap (Gu et al., 2016) enhanced for data pre-processing with the packages circlize (Gu et al., 2014) and matrixStats^6^ (Supplementary Table 4).

Class comparison was applied to a normalized matrix; gamma parametrization of means through a link function is impaired by negative values. The distribution of proteins was analyzed (Supplementary Figure 5).^5^ Variables were tested for normality and homoscedasticity (Supplementary Table 4, column BG). Parametric data were fitted using the identity link function and a GLM. Non-parametric data were fitted with an appropriate link function after calculating the point distance, using the raster R package,^7^ between exemplary fitted distributions and each response variable in our matrix (Supplementary Figure 5). There were two factors, root zone (two levels; tip and tip-adjacent) and treatments (four levels; control, cold, chitin, and chitosan). All interactions between factors were included. The control was the root zone tip-adjacent and the treatment “control.” There were eight regressors, i.e., β0 - Intercept, β1 - Factor 1 tip, β2 - Factor 2 chitin, β3 - Factor 2 chitosan, β4 - Factor 2 cold, β1 × β2 - Factor 1 tip:Factor 2 chitin, β1 × β3 - Factor 1 tip:Factor 2 chitosan, β1 × β4 Factor 1 tip:Factor 2 cold. Finally, *P* values were adjusted with the FDR proposed by Benjamini and Hochberg (1995) (FDR-BH95’; Supplementary Table 4).

Class prediction was applied to the same matrix used for class comparison. A combination of prediction methods with internal validation procedures and discriminant analyses based on least squares projections (PLS-DA or OPLS-DA) was used. Random forest used default settings of the R package randomForest (Wiener and Liaw, 2003); the classification error was plotted to decide which classes had good predictors. Two parallel tests were conducted, i.e., (1) between root zones and (2) cold against the other treatments. The importance of proteins as classification variables was evaluated using the Gini mean decrease. Simultaneously, the R package ropls (Thévenot et al., 2015) was used to fit an OPLS-DA to the root zone factor and a PLS-DA to the treatment factor. The variable importance for prediction (VIP) scores judged variable importance. Given that the latter analyses are more prone to overfitting, VIP scores were used as an interpretation aid (Supplementary Table 4).

## Results

**FIGURE 1:**
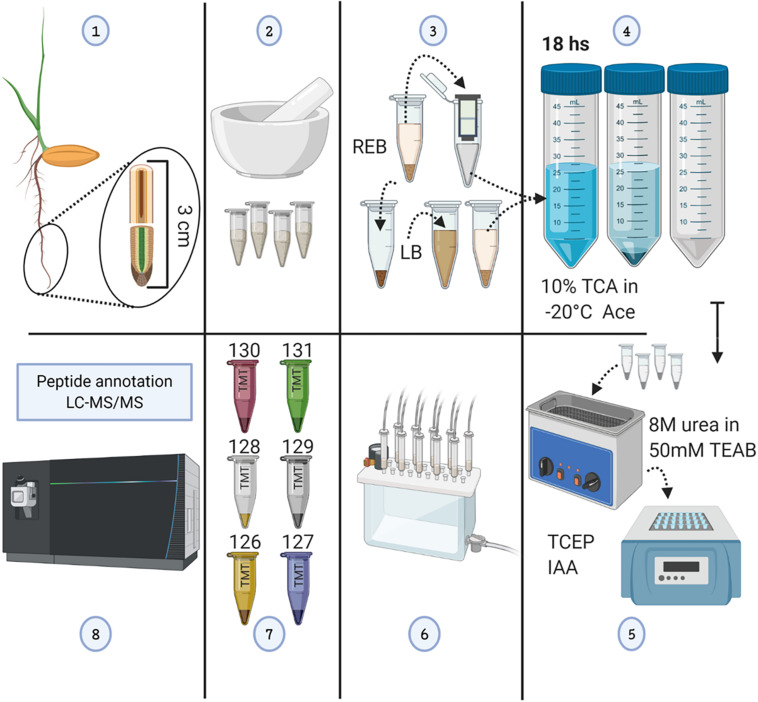
Methodological workflow to achieve measurements of total protein content and individual protein abundances in barley root tips. See also Supplementary Figure 1. **(1)** Harvesting of root tips from barley seedlings and division into two 1.5-cm segments. **(2)** Grinding of pooled tissue using liquid nitrogen, and mortar and pestle. **(3)** Proteome extraction; ribonucleoprotein complex enrichment using ribosome extraction buffer (REB) followed by detergent denaturation of the pellet using lysis buffer (LB). **(4)** Protein precipitation using -20°C cold 10% trichloroacetic acid (TCA) dissolved in acetone (Ace), subsequent centrifugation, and pellet washing. **(5)** Pellet solubilization, followed by protein reduction using tris(2-carboxyethyl)phosphine (TCEP; 10 mM of final concentration), alkylation with iodoacetamide (IAA; 55 mM of final concentration) in the dark, and overnight trypsin digestion. **(6)** Tryptic peptide cleaning using solid-phase extraction (SPE) cartridges. **(7)** Tandem mass tag (TMT) 6-plex labeling. **(8)** LC-MS/MS. The figure has been created with BioRender (https://biorender.com) and exported under a paid subscription.

Our sterile germination system avoided any light perception, allowing us to mimic optimal conditions during imbibition and early germination. After germination, barley seedlings were subjected to cold or mimic-biotic stimuli with fungal elicitors for a period of 5 days. This period of treatment-germination mimicked the natural time of seedling emergence, i.e., the time required for seeds when planted at a 50-mm depth to reach the soil surface (Kirby, 1993), while avoiding unnatural etiolation. Lack of etiolation was confirmed, and high-resolution images of the plants were taken (Supplementary Figure 1). Subsequently, the seminal root tips were harvested in two consecutive segments of 1.5 cm (Supplementary Figure 1), named as tip and tip-adjacent, and processed for proteome profiling (Figure 1). The tip sample contained the root cap, the meristematic cell division zone, the elongation zone, and parts of the early maturation zone without root hairs. The tip-adjacent zone contained the remainder of the maturation zone without lateral roots.

### Sampling the Barley Root Proteome

**FIGURE 2:**
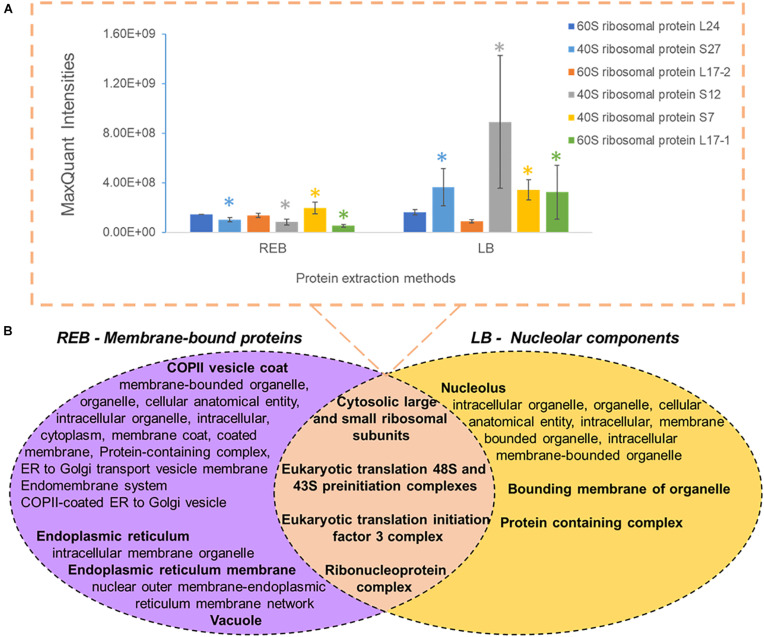
Comparison of unique and shared obtained barley proteome using two extraction methods (see also Supplementary Figure 2 and Supplementary Table 2), namely, a soft membrane solubilization using mild detergents [ribosome extraction buffer (REB)] and a hard membrane disruption using SDS [lysis buffer (LB)]. **(A)** Recovered Swiss-Prot *Hordeum vulgare* ribosomal proteins that were prioritized to control for the efficiency of different extraction methods (*n* = 3, error bars signal the standard deviation). Significance was determined per individual protein using a two-independent-samples *t*-test and is signaled by a colored star (*) when the *P* value was <0.05. **(B)** Comparison of different extraction methods sampling partially redundant and unique proteome fractions. Bold indicates the parent gene ontology (GO) term to the subsequent enriched cellular components according to a hierarchical sorting (Carbon et al., 2009; Mi et al., 2019). The enriched terms are interpreted as a group rather than individually. In the intersect, only translation-related GO parent terms are depicted; for the full categories, see Supplementary Table 2. Note that by combining both extractions, our methodology enriches membrane-bound mature ribosomes and pre-ribosomal particles present in the nucleolus.

We used “housekeeping” RP abundances as a proxy of ribosome abundance and protein synthesis. We gave priority to six RPs that were part of the manually curated entries in UniProt (Figure 2A). See Supplementary Table 1 for master annotation file. REB recovered significantly less protein of S7, S27, S12, and L17-1 as than did LB (Figure 2A). Oppositely, statistically equivalent protein abundances of L17-2 and L24 were recovered with both methods (Figure 2A). In general, raw protein intensities derived from identical amounts of roots extracted by equal buffer volumes were higher when using LB as compared with REB (Supplementary Figure 2). Moreover, lysis methods using REB or strong LB for plant root cells solubilized different and overlapping fractions of the proteome (Figure 2B).

We performed GO enrichment of cellular components using the Protein ANalysis THrough Evolutionary Relationships (PANTHER) classification system, followed by summarized semantic similarity-based plots obtained in REduce + VIsualize Gene Ontology (REVIGO) in order to understand the shared and specific proteomes between extraction methods (Supplementary Table 2). The strong extraction by LB, containing SDS, enhanced membrane disruption, allowing enrichment of nucleolar components. The milder detergent-mediated REB extraction solubilized membrane-bound complexes, cytoskeleton, or other insoluble proteins and protein complexes from transport vesicles, endoplasmic reticulum (ER), and Golgi components (Figure 2B). Consequentially, we combined the two extraction methods by performing first the native ribosome complex extraction via REB and then washing the pellet in denaturing LB buffer to recover the remaining proteome (Figure 1, step 3), thereby increasing proteome coverage while enriching for native ER-bound, nuclear and nucleolar associated ribosomes and RAPs.

### Induced Changes of the Root Proteome

#### Total Protein Contents

**FIGURE 3:**
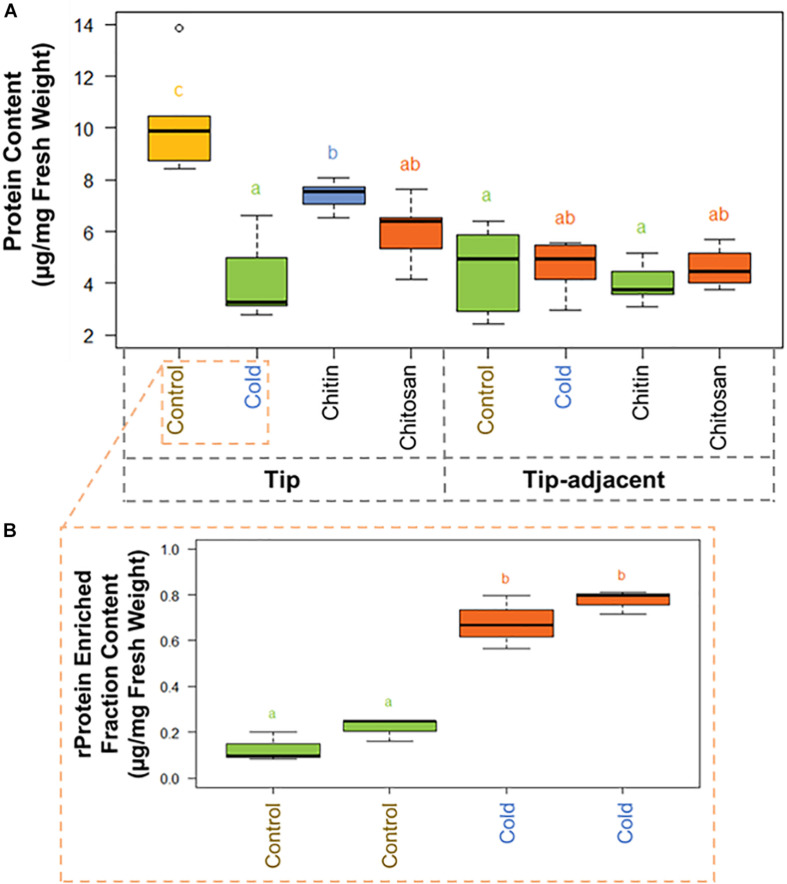
Protein content boxplots from *Hordeum vulgare* roots treated during germination with cold temperature, or additions to the germination solution (chitin or chitosan). See also Supplementary Figure 5 and Supplementary Table 3. **(A)** Total protein content was measured after protein extraction from 40 mg ± 5% of starting root material and overnight precipitation (*n* = 5). **(B)** Ribosomal protein (rProtein) content was measured after ribosome enrichment from 200 mg ± 5% of starting root material using a sucrose cushion and ribosomal protein dissociation using GuHCl as chaotrope (*n* = 3 × 2 experimental blocks). Protein contents were measured using the bicinchoninic acid (BCA) assay with starting tissue amounts that yielded protein concentrations in the linear range of the assay. The protein content values were calculated from a linear regression of bovine serum albumin (BSA) standards after blank correction [i.e., to 3 M of urea in 50 mM of triethylammonium bicarbonate (TEAB)]. Subsequently, we tested the data normality and homoscedasticity assumptions to assess the applicability of univariate statistical tests; we used the Shapiro–Wilk and the Brown–Forsythe Leven-type test based on the absolute deviations from the median, bootstrapped when *n* > 10. The tip and upper root zone datasets are normally distributed and homoscedastic; the full dataset is gamma distributed and homoscedastic (Supplementary Figure 5). Generalized linear models (GLMs) of the Gaussian and gamma families were used to evaluate the difference between the means in the datasets and gave equivalent statistical differences as an ANOVA comparison followed by Tukey honestly significant difference (HSD) test between means. Therefore, small letters and color progressions derived from the Tukey HSD test are used to denote statistically different means.

There was significantly more total protein in the root tip compared with tip-adjacent material (Supplementary Table 3). The tip was also significantly depleted in total protein more during cold treatments than when using elicitor treatments (Figure 3A). Conversely, the tip-adjacent zone did not undergo drastic protein content changes upon either of the treatments. We observed similar changes of the bulk proteome using Hawaii plots of our treated samples normalized to protein content and measured through LC-MS/MS (Supplementary Figure 3).

There were 2.12 times more protein in the tip segment with a mean value of 9.57 μg/mg FW. Cold treatment decreased the protein content significantly in the tip to 4.15 μg/mg FW, and the proteome composition differed markedly from control, implying that a reprogramming with less bulk protein occurs during the period of acclimation in root tips. Interestingly, proteome extracts enriched in ribosome complexes dramatically increased their content during cold acclimation (Figure 3B). The RP-enriched protein content increased from 2.6% (0.25 μg/mg FW) of the total protein in the control to 18% (0.75 μg/mg FW) of the total protein in the cold, suggesting a highly committed decision to either produce or not degrade ribosomes because the overall protein content drops significantly. By comparison, homeostasis of total protein content was retained in the tip-adjacent root zone during cold treatment with no significant change; however, many individual proteins were upregulated or downregulated. Mimicked-biotic stress treatments using chitin and chitosan elicitors significantly decreased the protein content to 7.39 and 6.00 μg/mg FW, respectively, only in the root tips and not in the tip-adjacent segments.

#### Individual Protein Abundances

**FIGURE 4:**
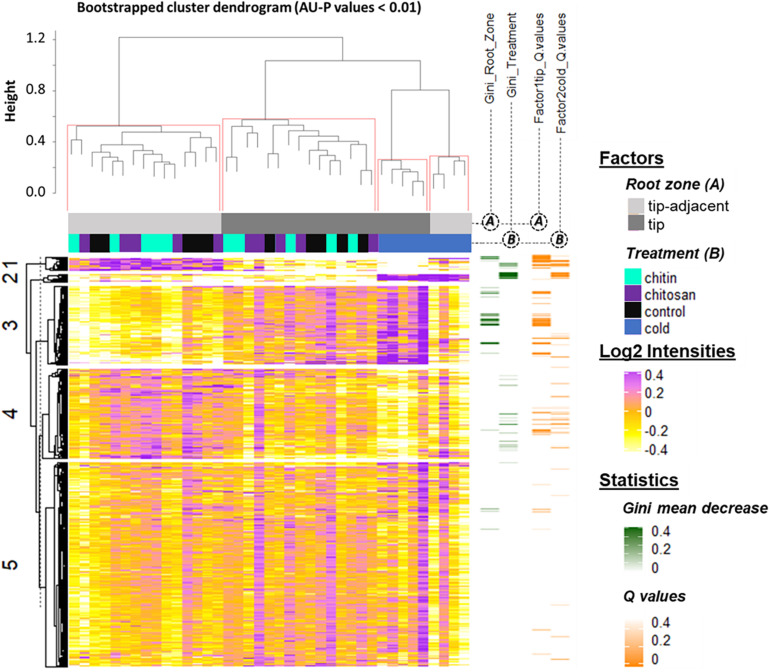
Summary log_2_-heatmap of statistical methods applied for class comparison, class discovery, and class prediction in LC-MS (*n* = 5). See also Supplementary Figures 3, 5, and Supplementary Table 4. Abscissa and ordinates were clustered using hierarchical cluster analysis (HCA) with Pearson correlation as distance measurement and average distance as clustering method. The clusters were bootstrapped, and approximately unbiased probability values (AU-*P*) values were calculated (red squares in the top cluster; see in Supplementary Table 4 the clustered ordinates). Additionally, to aid interpretation, K-means clustering was performed in the ordinates and indicated by numbers from 1 to 5 to the left side of the clusters. Response variables were tested for homoscedasticity and normality. Non-parametric tandem mass tag (TMT)-corrected protein intensity distributions from *Hordeum vulgare* roots (detailed in Supplementary Table 4) were evaluated (Supplementary Figure 5) by functions of the GitHub repository RandoDiStats (https://github.com/MSeidelFed/RandodiStats_package). Subsequently, an appropriate link function was selected to fit a generalized linear model (GLM). *P* values were adjusted into *Q* values using the FDR-BH95’. Orange colored rows in the third heatmap to the right indicate *Q* values < 0.05 in treatments that constituted a differential proteome. Finally, a random forest (Wiener and Liaw, 2003) analysis with default parameter settings was used to find markers suitable to predict the main factors of variance in the dataset, i.e., the classification of the two root zones and all treatments compared with cold. The green heatmap to the right contains the mean decrease Gini coefficient; a larger (dark-green) decrease means greater contribution of respective protein to predict sample classifications.

Statistical inference showed clear spatial and treatment-induced differences in proteome responses, validating previous observations and providing a proteomics fingerprint of the underlying biology (Figure 4 and Supplementary Table 4).

K-mean clustering of the ordinates found protein groups describing spatial root zones and treatments. Cluster 1 (Figure 4) featured proteins significantly increased in the tip-adjacent zone and significantly depleted during cold at an FDR Benjamini–Hochberg 1995 (FDR-BH95’) adjusted *P* values (aka Q values) < 0.05. Cluster 3 (Figure 4) contained proteins significantly increased in the root tips and/or during cold. Most protein markers of root zones and/or cold treatment, according to variable selection by random forest methodology, were found in these two clusters. The remaining cold predictors were in cluster 2, which featured the strongest cold-induced increases that were consistent in both tip-adjacent and tip segments. Cluster 4 contained proteins significantly depleted during cold and low abundant in root tips, similar to cluster 1 but with smaller intensity amplitudes. Cluster 5 featured only few significantly changed proteins. In order to prevent a single clustering solution to bias our statistical groups, we performed a bootstrapped HCA. The HCA was bootstrapped 10,000 times, and the results supported the same sample grouping. Briefly, approximately unbiased probability values (AU-*P* values) smaller than 1% from the bootstrapping analysis (Figure 4, red brackets) grouped separately the tip-adjacent root zone without the cold samples, the root tips without the cold samples, the cold-tip samples, and cold-tip-adjacent samples for a total of four significant groups. This means that the grouped samples would cluster together more than 99% of the times when clustering different sample proportions.

### Biological Context of Induced Protein Changes

**FIGURE 5:**
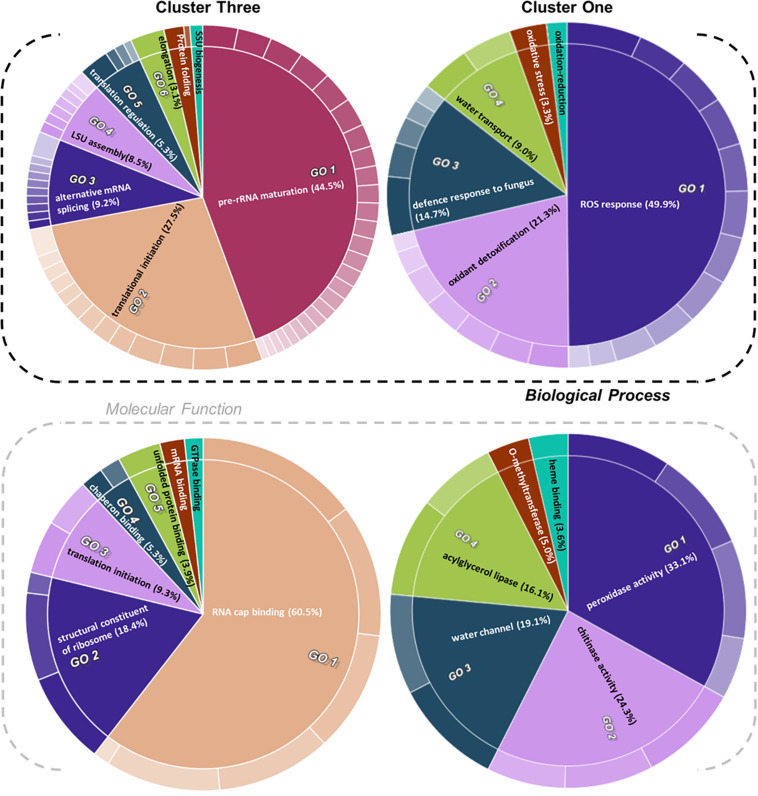
Gene ontology (GO) enrichment test of proteins from relevant clusters 3 and 1 of *Hordeum vulgare* roots (Figure 4). See also Table 1 and Supplementary Tables 5, 6. HORVU codes from significantly changed proteins were input into the gene ontology resource (Carbon et al., 2009; Mi et al., 2019) enrichment test. Subsequently, the lists of enriched GO terms with their false discovery rate (FDR)-adjusted *P* values were inputted into REVIGO (Supek et al., 2011) to aggregate redundant terms. Finally, input.csv files were generated and used to generate CirGO (Kuznetsova et al., 2019) (https://github.com/IrinaVKuznetsova/ CirGO) categorical plots. Intensity shades of colored categories reflect hierarchical relations between GO classes that were found enriched. Each subclass is represented in the plot by a white divisor line within each parent class, i.e., solid colors of the central pie charts. Note that GO-slim tests were performed. Complete cluster 1 was analyzed. Cluster 3 analysis was limited to a subset, i.e., Class 3 referred to in the text, which contained only the cold-modulated tip-accumulated proteins (interaction *P* values < 0.05 of Factor 1 tip:Factor 2 cold). The figure features Biological Process and Molecular Function GOs, Cellular Component GOs, and identities and names of all subclasses are reported in Supplementary Table 5.

We selected significantly modulated proteins from the clusters that characterized the spatial sampling and cold treatment for a GO enrichment analysis (Figure 5, see column AO of Supplementary Table 4). In a second, more stringent approach, we selected protein clusters that were supported by AU-*P* values < 0.01 (see column AP of Supplementary Table 4) for alternative GO enrichment analyses (Supplementary Table 5). To define significant changes, we fitted a GLM tailored to the distribution shape of each vector of protein intensities across treatments. When not parametric nor homoscedastic, the variance and mean components were parametrized with an appropriate link function by comparing with the square of skewness and kurtosis of average GLM distributions. The details of the statistical test are deposited and publicly available in a GitHub repository.^8^ Additionally, *P* values were adjusted for multiple testing using BH95’ to *Q* values (Figure 4, orange bars).

Cluster 1 contained proteins implicated in ROS production and plant defense (Figures 4, 5). In terms of biological processes, six categories were enriched: ROS response (49.9%), oxidant detoxification (21.3%), defense response to fungus (14.7%), water transport (9.0%), oxidative stress (3.3%), and oxidation-reduction (<1%). In terms of molecular function, peroxidase (33%) and chitinase (24%) activity hoarded more than 50% of the enrichments. The tip-adjacent region of barley root was well equipped in terms of defenses against environmental stresses compared with the younger root-tip tissue. Additionally, the lack of significant groupings of elicitor-treated plants (Figure 4) implies that priming the plants by exogenous chitin polymers did not induce a global-proteome reprogramming. A limited number of changes occurred. Proteins that play a role in fungal defense response were significantly decreased by chitin and chitosan treatments, for example, putatively annotated pathogenesis-related proteins (HORVU5Hr1G023720.1 and HORVU5Hr1G023740.2) and major pollen allergens with confident annotations (HORVU0Hr1G011720.5, HORVU4Hr1G054870.4, and HORVU7Hr1G034230.5), while chitinase-related proteins such as HORVU1Hr1G072250.1, HORVU7Hr1G113270.8, and HORVU7Hr1G121850.3 significantly increased following both priming conditions.

Cluster 2 contained cold-responsive proteins and the most relevant cold class predictors according to the Gini mean decrease of our random forest analysis. The cold response happened ubiquitously in both root zones, indicating a group of proteins that appears to be essential to the cold response in barley roots irrespective of spatial constraints. A GO enrichment test for biological processes revealed two categories: protein glutathionylation and glutathione metabolic process. Glutathione metabolism and *S*-glutathione conjugation upregulation included A hydroxyacylglutathione hydrolase-like protein, which produces glutathione [HORVU3Hr1G060920 (9 UP)]; a glyoxalase family protein, which produces glutathione [HORVU4Hr1G059270 (6 UP)]; and five different glutathione *S*-transferase family proteins [HORVU4Hr1G057890 (10 UP), HORVU1Hr1G049190 (11 UP), HORVU5Hr1G103430 (8 UP), HORVU4Hr1G057740 (16 UP), and HORVU6Hr1G026810 (13 UP)]. Additionally, this cluster contained 12 typical plant cold markers, COR/LEA proteins (Jaglo-Ottosen et al., 1998; Kume et al., 2005; Thalhammer and Hincha, 2013): HORVU6Hr1G064620 (13 UP), HORVU4Hr1G010750 (3 UP), HORVU3Hr1G066340 (7 UP), HORVU4Hr1G051780 (12 UP), HORVU2Hr1G099870 (11 UP), HORVU1Hr1G079280 (10 UP), HORVU1Hr1G056570 (7 UP), HORVU1Hr1G079290 (23 UP), HORVU4Hr1G074750 (31 UP), HORVU7Hr1G082040 (6 UP), HORVU5Hr1G092150 (2 UP), and HORVU6Hr1G083960 (19 UP); and finally, two abiotic stress markers, heat shock proteins, HORVU4Hr1G059260 (8 UP) and HORVU3Hr1G006530 (2 UP).

Proteins with significantly altered abundances from cluster 3 (Figure 4 and Supplementary Table 4) showed three types of responses: (Class 1) 60% of proteins (294/496) increased in the root tips, (Class 2) 11% of proteins (56/496) increased during cold, and importantly (Class 3) 29% of proteins (142/496) increased significantly in root tips after cold exposure. Class 3 contains 76 Class 1 and 19 Class 2 proteins and describes cold-modulated tip-accumulated proteins (interaction *P* values <0.05 of Factor 1 tip:Factor 2 cold). Considering that meristems are metabolic hotspots in active need of new proteins, we regarded Class 3 as potential holder of the newly synthesized proteome during cold. Hence, we focused on Class 3 for the following GO analysis. Class 3 revealed enrichment in translation-related processes and functions according to molecular function, biological process, and cellular component GOs (Figure 5 and Supplementary Table 5). Enriched terms from GO-slim analyses were hierarchically aggregated to reduce redundancy. In terms of biological processes, nine main categories were enriched, five related to translation or ribosome biogenesis: pre-rRNA maturation (44.5%), translational initiation (27.5%), ribosomal large subunit (LSU) assembly (8.5%), translation regulation, translation regulation (5.3%), elongation (3.1%), and ribosomal small subunit (SSU) biogenesis (<1%). Two additional categories were processes that feedback translation, namely, alternative mRNA splicing via spliceosome (9.2%) and chaperone-mediated protein folding (<1%). Molecular function analysis revealed seven enriched categories linked to the translational context: RNA cap binding (60.5%), structural constituent of ribosome (18.4%), translation initiation (9.3%), chaperon binding (5.3%), unfolded protein binding (3.9%), mRNA binding (<1%), and GTPase binding (<1%). Finally, cellular component analysis (Supplementary Table 5) yielded two categories related to ribosomes: cytosolic LSU (95.4%) and cytosolic SSU (4.6%).

Cluster 4 mimicked cluster 1 in terms of the direction of protein changes. Functionally, it contained biotic stress response components and enzymes that belong to the central metabolism. GO analysis did not reveal clear trends of enrichment patterns.

The functional categories enriched within statistically relevant clusters from K-means analyses outline the biological processes that were triggered in our dataset in response to the applied treatments. Nevertheless, the pattern of change of those categories across treatments remained unclear. Thus, we grouped protein responses per functional group in order to understand how those categories were changing in relative protein abundance across treatments (Table 1 and Supplementary Table 6). This allowed us to dissect the finer response of the elicitor treatments as compared with the global proteome reprogramming triggered by cold acclimation.

**TABLE 1:** Median protein intensity (log_2_ transformed, pooled-normalized) per ontology functional group sorted according to molecular function (clusters 1 and 3) or biological process (cluster 2).

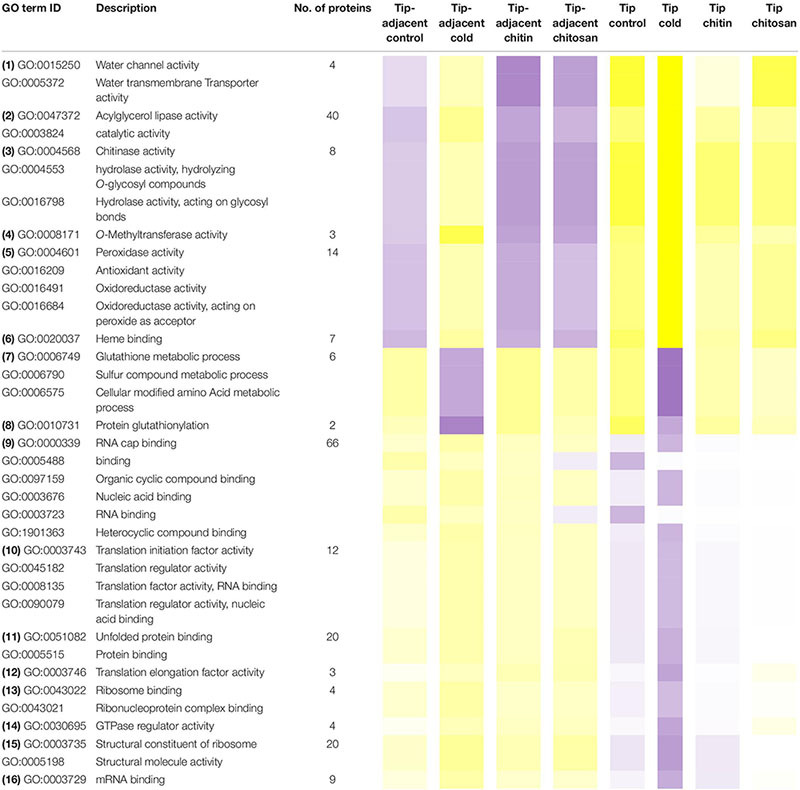

*Scale goes from -1 (yellow), through 0 (white) to 1 (purple).*

Functional categories related to cluster 1 (groups 1–6 in Table 1) are enriched in the tip-adjacent root segment while also depleted in this segment preferentially during cold. Additionally, the protein functional categories related to biological processes like ROS response, oxidant detoxification, and response stress to fungus are enriched in the elicitor treatments. The enrichment becomes evident analyzing boxplots of the mean response across treatments (Supplementary Table 6) and was not statistically evident before because the relative change of these proteins as compared with other treatments is rather small. A similar accumulation of the proteins comprising these functional categories happened in the more distal root-tip zone, where relative to the other treatments within the tip group these proteins increased in abundance during the elicitor treatments (shift from yellow in control to pale yellow/white in elicitor treatments). Biological process categories related to cluster 2 (groups 7 and 8 in Table 1) are majorly induced by cold across root zones with an additional interesting observation; i.e., glutathione metabolism is enriched in root tips, while protein glutathionylation is enriched in tip-adjacent segments. Functional categories related to cluster 3 (Class 3) (groups 9–16 in Table 1) are root-tip molecular markers that outline the accumulated proteome due to the cold response and are biologically related to the process of mRNA to protein translation. Noteworthy is that proteins within functional groups share mean responses with few outliers (boxplots in Supplementary Table 6), which implies that the chosen dissimilarity metric between protein abundances legitimately clustered responses across treatments.

Finally, we replicated the bootstrapped HCA, but this time using single proteins as variables. The HCA was bootstrapped 10,000 times using correlation as dissimilarity measure between proteins and average linkage as clustering method. We found protein sub-clusters supported by AU-*P* values <0.01 that indicated the tightest protein correlations across treatments. These typically small clusters of 2–12 proteins resided within all K-mean groupings (Supplementary Table 5). In cluster 3, we located three of these smaller high-confidence sub-clusters. Sub-cluster X109 consisted of nine proteins and contained chaperons functioning in ATP and unfolded protein binding that belong to the biological process, protein folding. Sub-cluster X151 consisted of five proteins from the process of ribosome biogenesis. Sub-cluster X1632 with 10 proteins contained GOs of membrane fission during mitotic cytokinesis, beta-glucan biosynthesis, and cell-wall biogenesis. Cluster 2 had a small sub-cluster X733 consisting of five proteins. This sub-cluster represented the same biological process as complete parent cluster 2, that is, glutathione metabolism and protein glutathionylation. Cluster 1 contained one sub-cluster, cluster X614 of 12 proteins that are implicated in enzyme-mediated hydrolysis by chitinase activity. Cluster 4 contained two small clusters: sub-cluster X904 with five proteins with functions in ubiquitin conjugating enzyme activity and sub-cluster X1347 with seven proteins, which are largely part of the proton-transporting V-type ATPase complex cellular component. Cluster 5 did not contain a high-confidence sub-cluster.

### Adjusting the Ribosomal Proteome During Cold Acclimation

Proteogenomics predicted open reading frame (ORF) redundancies are reduced in the high-confidence annotations provided by the barley consortium (Mascher et al., 2017). We used these annotations to sequence LC-MS/MS peptides and aligned those peptides to *Arabidopsis* RP paralogs. We report 68/80 RP families. Our alignment focused on the cytosolic ribosome-associated proteome, which shares a great extent of sequence similarity due to a common ribosome universal core (Bernier et al., 2018; Bowman et al., 2020). Additionally, the two aligned species are closely related metazoans, thus increasing the potential sequence similarity. Therefore, we used an algorithm based on Needleman–Wunsch (Needleman and Wunsch, 1970) global sequence alignment problem (Durbin et al., 1998; Haubold and Wiehe, 2006; Malde, 2008). This allowed us to interpret matching scores in a relative scale. A score of 10 was selected to filter out ambiguous annotations that map to multiple genes (Supplementary Table 7A).

Molecular markers of acclimated tips (Figure 4) were functionally related to multiple aspects of translation (Figure 5). Yet another group of translational-related proteins was significantly over accumulated in the tip (i.e., *P* values <0.05 for Factor1tip, Supplementary Table 4), but their abundances remained unchanged during cold (Supplementary Table 7B), suggesting that specific components from the translational machinery were modulated differently after the temperature shift. This prompted us to explore how the ribosome structural components and interacting factors were affected in barley roots upon a cold shift. We considered proteins related to translation when they included one of the translation-related GO terms or contained in their FASTA identifier the terms “translation,” “ribosome,” or “ribosomal.” In total, we found 269 translation-related proteins, including structural RPs, translation factors, and ribosome biogenesis factors mostly linked to cytosolic translation; 74% (198 of 269) of these proteins had a matching score higher than 10 to *Arabidopsis* homologs, which were further considered and included RAP and RP paralogs; 78% of the matched proteins (155 of 198) belonged to cluster 3, 20% (40 of 198) to cluster 5, and 1.5% (3 of 198) to cluster 4 (Supplementary Table 7).

**FIGURE 6:**
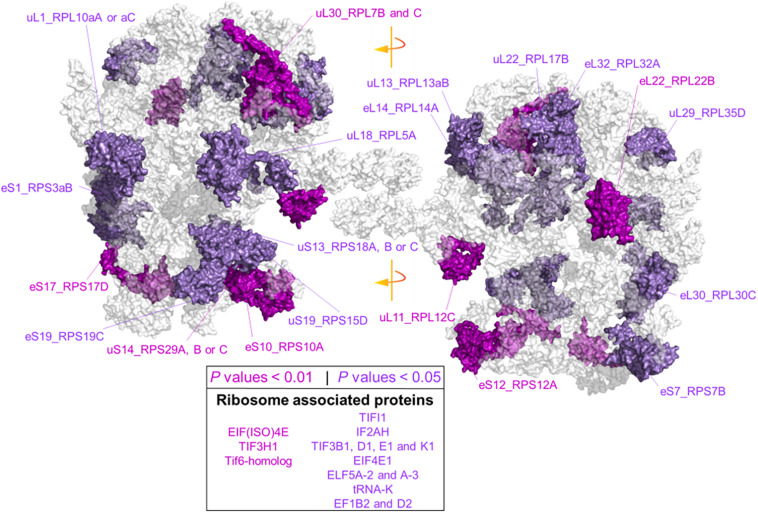
Status of the translational machinery in *Hordeum vulgare* roots after a shift to 4°C during 5 days of germination. See also Supplementary Table 7 and Table 2. Ribosome proteins (RPs) and ribosome-associated proteins (RAPs) were annotated by aligning amino acid sequences from LC-MS/MS barley annotations to the reviewed *Arabidopsis thaliana* proteome (Swiss-Prot, UniProt). The global alignment was performed in R using following the GitHub repository Align2FASTAs (https://github.com/MSeidelFed/Align2FASTAs). Only alignments with a minimum matching score of 10 were further analyzed. Changes in protein abundances across treatments were tested as detailed in Figure 4. RPs and RAPs with significantly increased abundances at *P* values <0.05 (dark magenta) or <0.01 (purple) during the cold treatment in roots (i.e., interaction *P* values <0.05 of Factor1tip:Factor2cold) were mapped into a 2D rendering of a 3D Cryo-EM model of the wheat translating ribosome. RAPs, e.g., translation initiation factors and ribosome biogenesis factors with significant changes (Supplementary Table 7), are reported in the inserted box.

Due to our protein extraction method, the displayed changes were an average of all mature, immature, translationally competent, and translationally inactive “reserve” ribosomes in the cell. In plants, there are 80 cytosolic RP families, and *Arabidopsis* features two to seven paralog genes of each RP (Barakat et al., 2001). In our study, after aligning the protein sequences of the barley translational machinery with the *Arabidopsis* reviewed proteome (Swiss-Prot, UniProt), we found barley representatives from 74 cytosolic and two mitochondrial RP families. We found unique peptides and used them to define 85 certain RP paralog matches. After including matches from non-unique peptides, we defined 101 potential RP paralogs (Supplementary Table 7C). Abundances of 22 paralogs significantly differed during cold in the root tips (Figure 6 and Supplementary Table 7). We mapped the significant changes onto a 2D projection derived from a 3D Cryo-EM structural model of the wheat translating monosome (Armache et al., 2010) in order to understand the spatial distribution of the cold-induced changes. This allowed us to obtain an overview of total RP shifts relative to the complex structure that happened in barley root tips at the onset of cold acclimation (Figure 6).

Besides structural RPs, we assessed several RAPs that transiently bind and assist translation at different stages and were modulated in the root tips by cold in our dataset. Eleven translation initiation factors (TIFs), one maturation factor homolog (i.e., Tif6-homolog), one tRNA ligase (lysine), and two elongation factors (i.e., elongation factors 1 β2 and δ2) had significantly different abundances during cold in the root tips (Supplementary Table 7 and Figure 6). All the *P* values that belong to cold enrichments outlined in Figure 6 have been compiled and extended in Table 2. The extended version in Table 2 includes *H. vulgare* identified protein peptides, *A. thaliana* homolog matches with their respective match score, and RP or RAP paralog identities.

**TABLE 2:** Statistics of cold acclimated and homology alignments of significantly changed-*Arabidopsis thaliana* (AT) and *Hordeum vulgare* (HORVU) RP and RAP paralogs outlined in Figure 6.

**Paralog**	**AT code**	**Match score**	**HORVU Code**	*P* value*
**eS10_RPS10A**	AT4G25740	135.3	HORVU0Hr1G004700.1	0.003
**uS14_RPS29A B, or C**	AT3G43980,AT3G44010,AT4G33865	192.0	HORVU5Hr1G098740.1;HORVU0Hr1G011940.3;HORVU4Hr1G037800.1	0.003
**uS14_RPS29A, B, or C**	AT3G43980,AT3G44010,AT4G33865	181.3	HORVU5Hr1G098740.1;HORVU0Hr1G011940.3;HORVU4Hr1G037800.1	0.003
**EIF(ISO)4E**	AT5G35620	22.9	HORVU1Hr1G039260.2	0.003
**eS17_RPS17D**	AT5G04800	41.5	HORVU1Hr1G042220.1	0.003
**uL30_RPL7B**	AT2G01250	275.0	HORVU2Hr1G100970.4	0.003
**AtTIF3H1**	AT1G10840	563.2	HORVU2Hr1G068360.8	0.005
**eS12_RPS12A**	AT1G15930	93.0	HORVU2Hr1G053290.6	0.006
**eL22_RPL22B**	AT3G05560	210.1	HORVU2Hr1G019160.1	0.008
**uL11_RPL12C**	AT5G60670	414.9	HORVU6Hr1G067870.1	0.008
**Tif6-homolog**	AT3G55620	715.5	HORVU2Hr1G026220.5	0.009
**IF5A2**	AT1G26630	364.9	HORVU2Hr1G063210.2	0.010
**eL30_RPL30C**	AT3G18740	127.4	HORVU0Hr1G023290.1;HORVU1Hr1G068640.1	0.012
**eL30_RPL30C**	AT3G18740	74.2	HORVU0Hr1G023290.1;HORVU1Hr1G068640.1	0.012
**IF2AH**	AT2G40290	641.6	HORVU2Hr1G014580.1	0.013
**IF5A3**	AT1G69410	371.2	HORVU2Hr1G034400.4	0.014
**EF1D2**	AT2G18110	246.3	HORVU2Hr1G031450.6	0.015
**EF1B2**	AT5G19510	299.6	HORVU2Hr1G022090.4	0.015
**eS7_RPS7B**	AT3G02560	296.6	HORVU1Hr1G029870.1	0.017
**uL13_RPL13aB**	AT3G24830	103.2	HORVU2Hr1G063880.1	0.018
**AtTIFI1**	AT2G46280	573.0	HORVU4Hr1G040280.4	0.019
**tRNA-K**	AT1G69410	663.9	HORVU5Hr1G106380.6	0.021
**AtTIF3E1**	AT3G57290	303.7	HORVU5Hr1G072560.1	0.024
**AtTIF3K1**	AT4G33250	238.0	HORVU4Hr1G077250.1	0.024
**uL18_RPL5A**	AT3G25520	284.8	HORVU2Hr1G073320.1	0.027
**AtTIF3B1**	AT5G27640	965.0	HORVU5Hr1G106440.1	0.028
**uL29_RPL35D**	AT5G02610	282.0	HORVU2Hr1G068120.2	0.029
**uL22_RPL17B**	AT1G67430	419.0	HORVU7Hr1G054010.1	0.031
**IF4E1**	AT4G18040	74.1	HORVU3Hr1G113940.1	0.031
**eS1_RPS3aB**	AT4G34670	548.5	HORVU4Hr1G070370.1	0.033
**eL32_RPL32A**	AT4G18100	264.9	HORVU5Hr1G075420.2;HORVU5Hr1G075500.3	0.034
**eL32_RPL32A**	AT4G18100	262.2	HORVU5Hr1G075420.2;HORVU5Hr1G075500.3	0.034
**uS4_RPS9C**	AT5G39850	553.3	HORVU5Hr1G038920.1	0.039
**eS19_RPS19C**	AT5G61170	146.3	HORVU5Hr1G052240.6	0.040
**uS19_RPS15D**	AT5G09510	63.4	HORVU2Hr1G057430.1	0.041
**uS13_RPS18A, B, or C**	AT1G22780,AT1G34030,AT4G09800	332.0	HORVU5Hr1G104720.1	0.043
**uL1_RPL10aA**	AT1G08360	624.7	HORVU7Hr1G059090.5;HORVU4Hr1G001350.5	0.045
**uL1_RPL10aC**	AT5G22440	246.8	HORVU7Hr1G059090.5;HORVU4Hr1G001350.5	0.045
**eL14_RPL14A**	AT2G20450	335.3	HORVU6Hr1G058560.1	0.046
**uL30_RPL7C**	AT2G44120	465.0	HORVU5Hr1G108990.2	0.047
**AtTIF3D1**	AT4G20980	400.8	HORVU1Hr1G088760.2	0.049

*P values of the interaction term of cold and root-tip factor, i.e., β1 × β4. Underlined duplicated HORVU and their respective AT entries are explained in Supplementary Table 7B.RP, ribosomal protein; RAP, ribosome-associated protein.

Finally, we found additional clusters of RPs with high probability support (AU-*P* value < 0.01 in Supplementary Table 7). We considered those sub-clusters of four or more RPs as groups having strong co-dependence: sub-cluster X151 with SSU-RP eS7_RPS7B and LSU-RPs uL22_RPL17B, S1_RPS3aB, eL6_RPL6C, and uL18_RPL5A. These proteins were significantly more abundant during cold and good molecular markers of root tip characterized by a large Gini mean decrease: cluster X471 with SSU-RPs eS6_RPS6B, eS24_RPS24A, and uS17_RPS11C and LSU-RPs uL5_RPL11A, eL34_RPL34A, and eL13_RPL13B. Proteins within these two sub-clusters were significantly more abundant in the root tips. The top five molecular markers according to the mean decrease of Gini ranking among RPs of the root tip were eL8_RPL7aA, eS28_RPS28A or B, uS2_RPSaA, uS7_RPS5A, and eL14_RPL14A; as for roots acclimated to cold, the best and only RP marker was eS26_RPS26C.

## Discussion

### Translational Reprogramming

During cold acclimation, total protein contents relative to fresh root weight drop in root tips, while a fraction enriched in ribosome-bound protein increases its protein content dramatically. Thus, in spite of reducing significantly total protein, a highly committed decision to either produce *de novo* or retain old ribosomes is made by the plant. A second, MS, piece of evidence shows that specific proteins, in samples normalized to protein content, significantly increase their relative abundance in barley root tips during cold exposure. GO analysis revealed that those proteins were involved in protein synthesis. The results range from ribosome biogenesis, structure, to mRNA-splicing, recruiting, translation initiation, elongation, and protein folding, suggesting that specific components of the translation machinery need to be accumulated to cope with cold. This fits into what is known for *Arabidopsis* protein translation apparatus, which is a central hub mediating responses to cold acclimation (Beine Golovchuk et al., 2018; Garcia-Molina et al., 2020). Interestingly, RAPs and RP paralogs differ in their amino acid sequence from barley to *Arabidopsis* homologs with varying degrees (Supplementary Table 7). Scores indicated that multiple barley RP paralogs match differently to the same *Arabidopsis* family (e.g., uL3_RPL3A and uL1_RPL10aA). The ranges in matches indicate sequence and potential functional divergence of paralogs. Moreover, cold-induced changes in specific paralogs suggest that the composition of barley cold-acclimated ribosomes differs from a canonical complex, and during cold, there might be a paralog-specialized ribosome (Gerst, 2018; Samir et al., 2018; Segev and Gerst, 2018; Ghulam et al., 2020). Additionally, the abundance of many RAPs, especially TIFs, increased. If there is a temperature-induced slowing-down of both translation initiation and elongation, and the former is being slowed down more than the latter, the expectation is an increase of 80S, 48S, and 43S complexes relative to a decreased amount of polysomes, which could contribute to the increased amount of TIFs during cold acclimation.

Interpretation of shifts in individual translational components must be contextualized to the cell lysis and protein extraction methods. Solubilization differences in control RPs argue that the nucleolus is recovered when using LB extraction (Figure 2B). Ribosome biogenesis in eukaryotes starts in the nucleolus (Woolford and Baserga, 2013; Baßler and Hurt, 2019; Sáez-Vásquez and Delseny, 2019; Martinez-Seidel et al., 2020); thus, many pre-ribosomal complexes at different stages coexist inside. Non-translational particles may change their RP stoichiometry as compared with translationally competent complexes upon temperature changes (Cheong et al., 2021), suggesting that a cold-specific ribosome biogenesis could give rise to specialized ribosomes in plants (Ohbayashi and Sugiyama, 2018; Martinez-Seidel et al., 2020). Thus, solubilizing the nucleolus implies that, first, our results indicate a total average status of translational protein components and, second, they might signal essential RP-mediated mechanisms of cold-specific ribosome biogenesis.

Shifts in the plant proteome occur during cold acclimation. Several possibilities could contribute to such shifts. For instance, through a cold-specialized ribosome population, the root-tip cells could direct translation toward a subset of alternatively spliced transcripts (Thompson et al., 2016). Alternative splicing is a major response to cold in *Arabidopsis* (Calixto et al., 2018), and now we have shown that protein components of the spliceosome need to be accumulated as a cold response in barley roots (Figure 5 and Supplementary Table 5, Biological Process, cluster 3). The activation of the spliceosome happens in root tips only, suggesting a correlation to cold-accumulated ribosomes and specific ribosome components. Interestingly, the Partner of Y14 and Mago (PYM) protein factor was drastically accumulated during cold (Cluster 2, Figure 4). In yeast, PYM is anchored to the 48S preinitiation complex and is able to promote translation of spliced mRNAs (Diem et al., 2007) by interacting with the exon junction complex (EJM), which is a cellular shuttle for spliced transcripts from the nucleus to the cytosol (Boisramé et al., 2019). Recruiting of spliced transcripts could be one of multiple layers complementing an altered ribosomal proteome to achieve selective translation and rapid proteome shifts during cold. Alternatively, cold acclimation has been shown to trigger transcriptional reprogramming events in cereals that lead to nucleosome and chromatin remodeling (Janská et al., 2011). These events could tailor the translated proteome by changing the transcriptional dynamics of cold-responsive genes and eventually altering the transcript substrate availability for ribosomes to translate. These strategies are not mutually exclusive and might act independently but converge on a proteome shift or act in concert to achieve selective transcript translation and rapid proteome shifts during cold acclimation.

Freezing tolerance and cold stress memory are triggered by the same stimuli but might be executed to completion by different cellular machinery. Freezing tolerance is partly achieved by induction of the CBF regulon and its subsequent modulation on the COR/LEA signal transduction pathway in plants (Jaglo-Ottosen et al., 1998). Plant reports of this correlation include cereals such as wheat (Kume et al., 2005; Sasaki et al., 2014) or rice (Wang et al., 2016; Xiao et al., 2018). In wheat, there is an accumulation of COR/LEA proteins in the crowns during cold acclimation (Houde et al., 1995), and, as we now report, these proteins accumulate in actively dividing barley root meristems during cold acclimation. The same link between the CBF regulon and a posterior COR/LEA upregulation is established in *Arabidopsis* (Hundertmark and Hincha, 2008). Cold stress memory in *Arabidopsis* is maintained for 7 days and partly depends on induction of lipid, secondary metabolism, and stress or growth-related functions (Zuther et al., 2019), depending on how cold-tolerant the accession is. Typical cold-responsive genes such as the CBF regulon or COR/LEA protein coding genes are less implicated in cold stress memory (Zuther et al., 2019; Leuendorf et al., 2020). Thus, cold stress memory could make use of a primordial cellular machinery to conserve a recording of the stimulus. In this context, an altered ribosomal composition may act as molecular memory of the initial cold cue. This seems logical considering that ribosomes have a typical half-life of 3–4 days in plants (Salih et al., 2019), which might be stretched due to the slower cold molecular dynamics. Thus, to prevent the high costs of triggering an appropriate response from scratch upon new stress cues (Van Hulten et al., 2006), it is likely that an altered ribosome composition is stored as molecular memory.

### Acquired Cold Tolerance

Priming, in the context of plant stress, is defined as a trigger cue that increases the future performance of plants responding to stress (Hilker et al., 2016). To achieve the primed state, plants must improve the overall stress management, strengthening self-defense systems to defy environmental cues (Hilker and Schmülling, 2019). Upon specific cues, plants change epigenetic patterns, accumulate transcriptional factors, and modify the expression levels of genes and accumulation of proteins, including PTM status and metabolites (Hilker et al., 2016; Hilker and Schmülling, 2019). Our study evidenced the intrinsic enrichment in root tip-adjacent zones of ROS response, oxidative detoxification, and fungal defense proteins. Many of these possess pathogen-resisting enzymatic activities, e.g., peroxidase and chitinase. Nevertheless, many of these proteins do not dramatically respond to pathogen-associated molecular patterns (PAMPs) such as chitin and chitosan. Thus, general response patterns and molecular consequences from a pathogen attack in the root tips must stem from other components of the stress.

Acquired cold tolerance for later biotic stress in plants is attributed to the elements in common that both the stresses have (Ben Rejeb et al., 2014). First, a common group of transcription factors (TFs) such as the mentioned cold-specific CBF factors or the MYB, NAC, and DREB factors. These TFs enable a cross-talk between abiotic and biotic stresses by modulating pathogen resistance (PR) genes and their proteoforms (Snider et al., 2000; Tsutsui et al., 2009; Seo and Park, 2010; Seo et al., 2010). PR proteins function for disease resistance and are also implicated in protecting the plant during overwintering (Kuwabara and Imai, 2009). Second, during the plant cold response, the first shared elements with responses to biotic stress are ROS signaling and calcium influxes (Ben Rejeb et al., 2014). The oxidative component of both stresses produces an oxidized cellular status (Pastor et al., 2013). This state could be compensated for, especially at the proteome level, to avoid ROS-derived damages.

Our results offer testable hypotheses that could form a solid mechanistic link between cold acclimation and cold-induced plant defense against pathogens (Kuwabara and Imai, 2009; Szechyñska-Hebda et al., 2013; Szechyńska-Hebda et al., 2015; Sasaki et al., 2016). Defense-triggered cold processes can be divided into two in our dataset. First, proteins in the tip-adjacent root zone are functionally related to defense and are both depleted during cold and accumulated as a functional group during the elicitor treatments. This implies that elicitor-derived resistance relies on the upregulation of a defense-related cellular machinery to confer a primed state while cold-acquired resistance makes use of a different cellular machinery. Second, GO analyses revealed that the machinery involved in synthesis and assemblage of glutathione as a PTM is upregulated in both root zones. *S*-Glutathionylation shields against oxidative stress by acting as a ROS scavenger (Diaz-Vivancos et al., 2015). Thus, it seems likely that cold-acquired resistance does not rely on upregulating the plant immune system protein components. Rather, we argue that chilling temperature could prepare plants to withstand oxidative stress, thus providing the means to resist forthcoming cold-related diseases, presumably caused by winter or fungal pathogens. Due to its ubiquitous upregulation, all proteins are likely being shielded by *S*-glutathione during a cold cue. Hence, we argue that resistance for a later pathogenic fungal interaction may be gained because the accumulated ribosomes or any other proteins that are newly synthesized would be less affected by the oxidative component of biotic stress. Methodological steps employed for proteomics analysis using global reduction and latter alkylation of reduced thiols precludes us to distinguish between reduced cysteine from S–S disulfide bonds, free thiols, or *S*-glutathionylation. Thus, it would not be possible to find in our current dataset *S*-glutathionylated peptides. These additional experiments with dedicated methods and method developments are now motivated by our findings. At which point, it will become evident what portion of the proteome is actually protected by the PTM.

Finally, after a thorough search of the fungal-elicitor-induced proteome changes, we did not find global proteome modulatory effects. Rather, the observable shifts happened when specific defense-related proteins were pooled into functional groups and analyzed as a combined mean response. Hence, we constrained the search to enzymes that are directly related to chitin polymers and could be measured. Our results indicate that chitin polymers modulate the plant defense system without triggering major proteome rearrangements in the host plants. Comprehensive effects of chitin naturally occurring polymers on barley seedlings along with a precise measurement of the proteins involved in the responses still need further investigation.

### Summary

Our results indicate that cold acclimation triggers a drop in protein content in barley root tips, while specific proteins are accumulated. We found specific accumulation of cold markers such as COR/LEA proteins that also accumulate in mature tissues such as cereal crowns during cold. We couple these changes to a highly committed and drastic ribosome accumulation, which could imply the assembly of cold-rewired ribosomes characterized by substoichiometric RP compositions. Substoichiometry can arise from the significant changes in relative abundances of specific RP paralogs found in our study. Divergent ribosomes are further supported by the accumulation of spliceosome components in root tips, which could tailor an alternatively cold-spliced transcriptome that would rely on selective translation. These regulatory mechanisms, acting upon the proteome, can be amplified using root meristems as a model to study rapid proteome reprogramming. We exemplify the accumulation of proteins involved in *S*-glutathione biosynthesis and *S*-glutathione conjugation as indicators of *S*-glutathionylation PTM of proteins during cold acclimation and put it forward as a new hypothesis on which to build future studies of cold-acquired tolerance linked to defense and protein synthesis. Finally, in spite of the gained advantages of using only root tips instead of complete root systems to identify proteome shifts, finer spatial resolution will be needed to allow discriminating between all the biological steady states coexisting in apical root zones.

## Data Availability Statement

The datasets presented in this study can be found in online repositories. The names of the repository/repositories and accession number(s) can be found below: http://proteomecentral.proteomexchange.org/cgi/GetDataset, PXD021731.

## Author Contributions

FM-S and PS performed the experimental work. SN, ML, and NW contributed in LC-MS/MS. FM-S, PS, and AAPF contributed in the protein extraction. FM-S, PS, ML, and BB contributed in the conceptualization and production of *in silico* resources. UR, JK, and BB contributed in the conceptualization, experiment planning, and manuscript editing. FM-S, PS, and BB contributed in the manuscript writing and figure designing. All authors contributed to the article and approved the submitted version.

## Conflict of Interest

The authors declare that the research was conducted in the absence of any commercial or financial relationships that could be construed as a potential conflict of interest.

## Footnotes

1 http://pgsb.helmholtz-muenchen.de/plant/barley/index.jsp

2 https://github.com/MSeidelFed/Align2FASTAs

3 https://www.rdocumentation.org/packages/Biostrings/versions/2.40.2/topics/pairwiseAlignment

4 https://blast.ncbi.nlm.nih.gov/Blast.cgi?PAGE_TYPE=BlastSearch&PROG_DEF=blastn&BLAST_PROG_DEF=blastn&BLAST_SPEC=GlobalAln&LINK_LOC=BlastHomeLink

5 https://github.com/MSeidelFed/RandodiStats_package

6 http://CRAN.R-project.org/package=matrixStats

7 http://CRAN.R-project.org/package=raster

8 https://github.com/MSeidelFed/RandodiStats_package

## Abbreviations

AU-*P*: approximately unbiased probability values
BSA: bovine serum albumin
FDR: false discovery rate
FW: fresh weight
GLMs: generalized linear models
LB: lysis buffer
PTM: post-translational modification
RAP: ribosome-associated protein
REB: ribosome extraction buffer
ROS: reactive oxygen species
RP: ribosomal protein
SC: sucrose cushion
S-GSH: *S*-glutathionylation
TMT: tandem mass tag
