# Supplementary material for: Membrane-Enriched Proteomics Link Ribosome Accumulation and Proteome Reprogramming With Cold Acclimation in Barley Root Meristems
Source: Front Plant Sci. 2021 Apr 30;12:656683. doi: 10.3389/fpls.2021.656683 (PMC8121087; doi:10.3389/fpls.2021.656683)
Supplement: Supplementary file 8 [file Data_Sheet_1.DOCX]

Supplementary Material

# Supplementary Figures


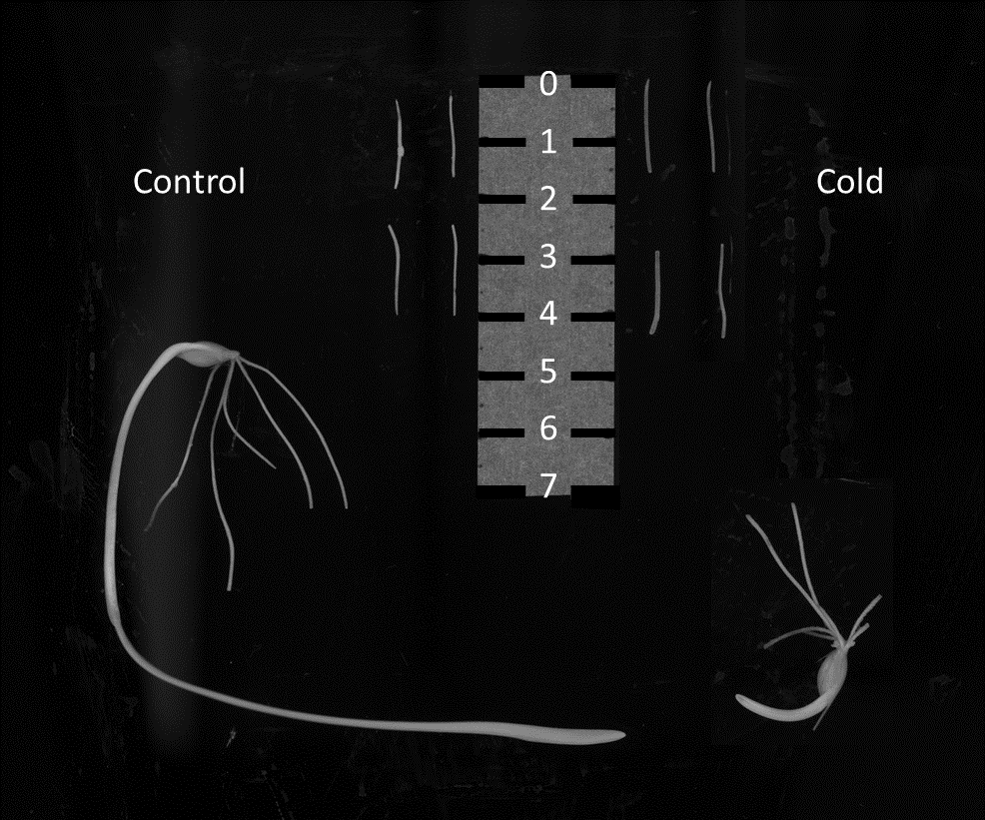


**Supplementary Figure 1. *Hordeum vulgare* harvested root tip segments. Related to Figure 1 - step 1.** The scale contains centimeters from 0 to 7. The left part of the scale features two root tips separated in two 1.5 cm segments each, and one barley seedling reared at a control temperature of 25°C for 16 h and 18°C for 8 h, daily temperature fluctuation in the dark. The right part of the scale contains two root tips separated in two 1.5 cm segments each, and one barley seedling shifted to 4°C after 48 hours of germination. Both plants feature ~ five seminal roots that were already removed when the image was taken and only two of them used for the image. The seminal root tip (~ 0 – 1.5 cm) and tip-adjacent (~ 2.5 – 4 cm) zones are contiguous at both sides of the scale.


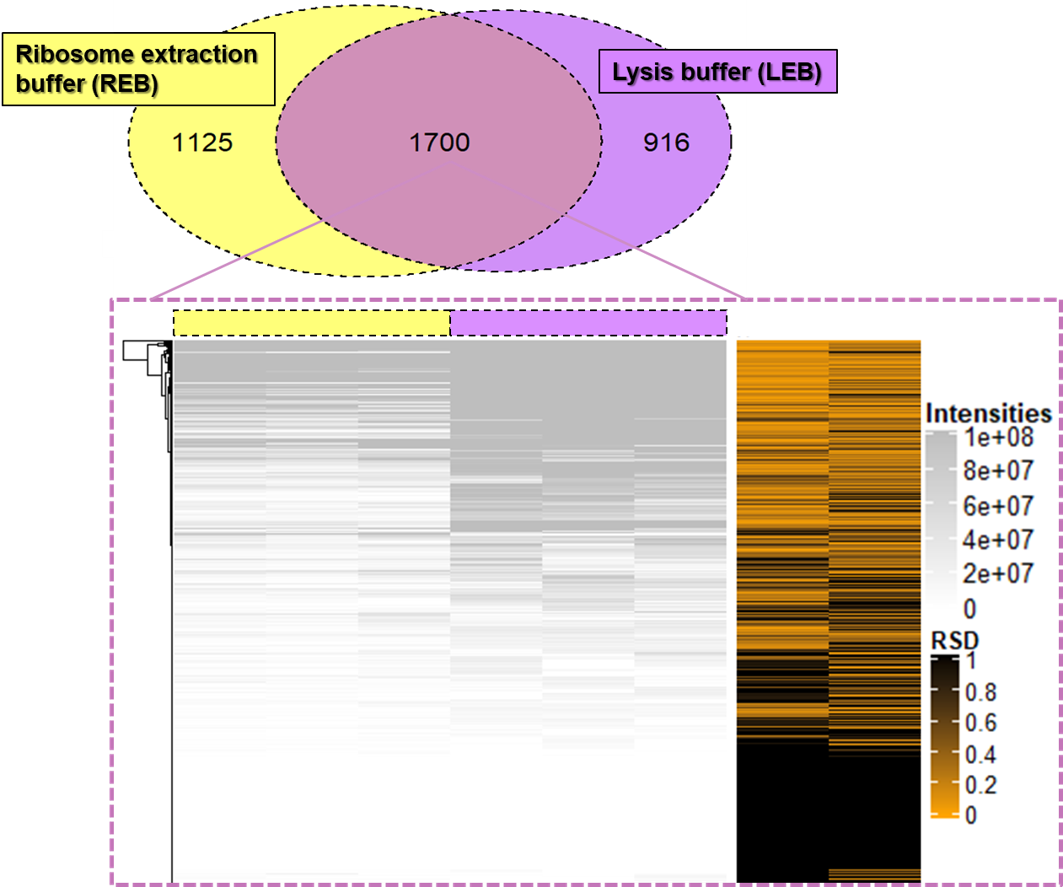


**Supplementary Figure 2.** **Intensities of common proteins measured through two independent extraction methods from barley root tips. Related to Figure 2.** Peptides from raw LC-MS/MS chromatograms were annotated using MaxQuant and FASTA sequences compiled in **Supplementary Table 1**. Subsequently the protein identifiers were used to intersect both extraction methods yielding 1700 proteins in common. The abundances of common proteins were used as input for this Heatmap using the ComplexHeatmap R package (Gu et al., 2016) without any transformation or normalization. Three independent replicates were used to calculate the relative standard deviation (i.e., RSD orange - black Heatmap) per individual protein. Note that lysis buffer increases the intensities from the common proteins as compared to ribosome extraction buffer. Nevertheless, the relative standard deviations do not differ substantially between both extraction methods.


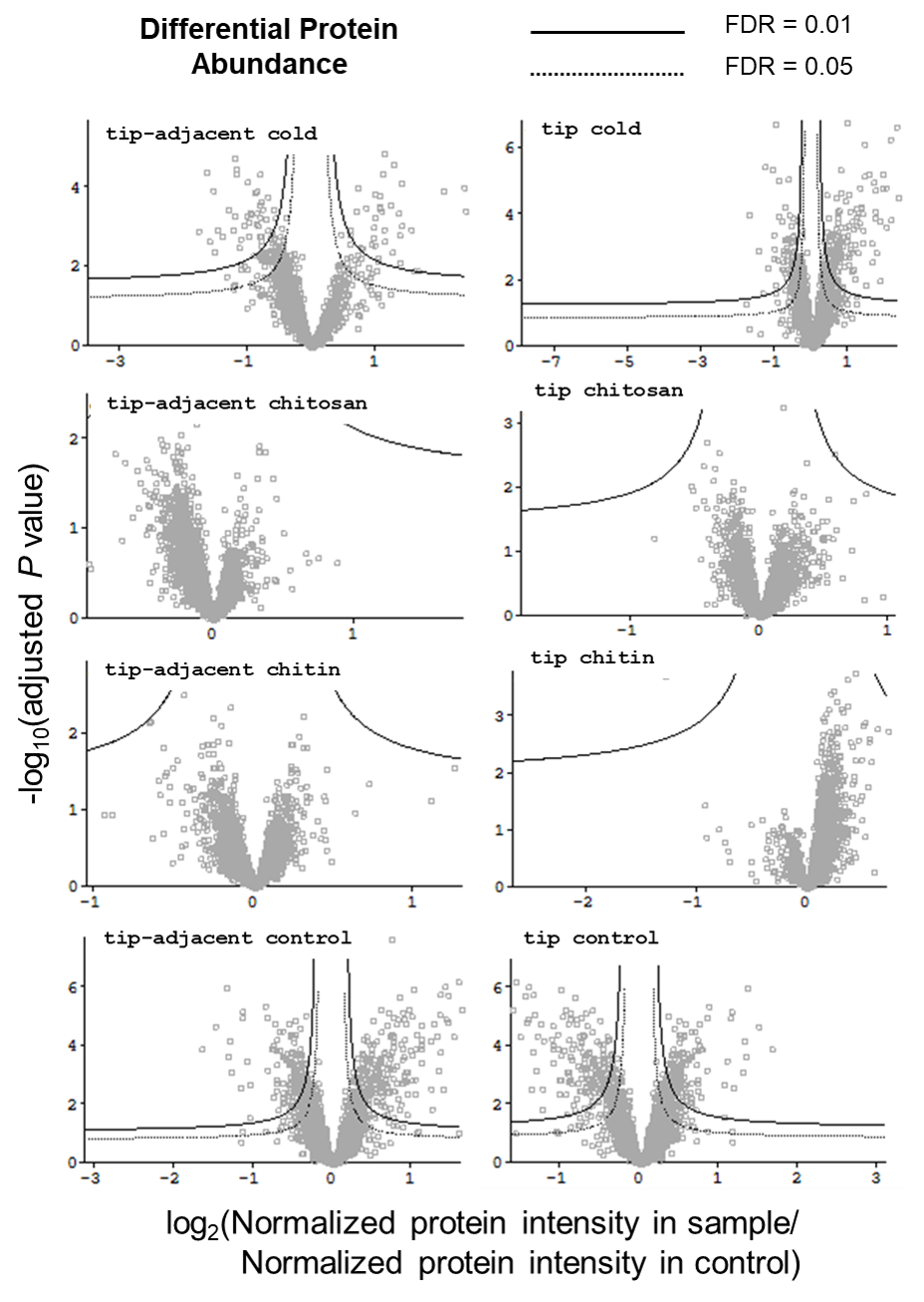


**Supplementary Figure 3.** **Hawaii plot of *Hordeum vulgare* root proteome subjected to different treatments of mimicked-biotic or abiotic stress. Related to Figure 4.** RAW chromatograms of TMT labelled samples were processed with MaxQuant using the FASTA sequences reported in **Supplementary Table 1**. Subsequently, the protein group matrix was inputted into Perseus (v 1.6.12.0) for adequate pre-processing, where a compendium of pooled samples was used to normalize the intensities of all samples and enable legitimate cross comparisons. A 1% FDR filter was applied using reverse hits in order to decrease the false annotations. Finally, only protein groups with more than one of razor + unique peptides were used. In the plot, the tip-adjacent and tip root zone controls have been used as divisor for their respective treatment ratios, as well as for the other zone controls. Hence, values in x-axes of the volcanoes represent a log_2_-transformed ratio of normalized protein intensity in each sample over that in the control. Note that root zones feature a significantly different proteome beyond the FDR = 0.01 barrier as compared to one another. Within treatments, cold features many proteins that differ significantly from its control while only a couple of proteins were significantly regulated in chitin and chitosan treatments.


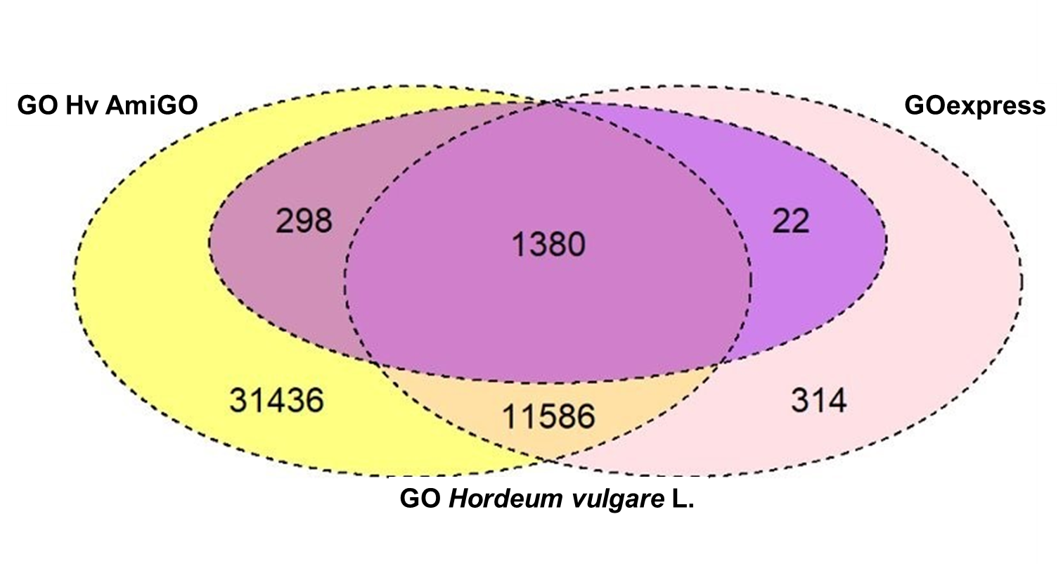


**Supplementary Figure 4.** **Sources used to obtain the full set of GO terms.** **Related to Supplementary Table 1.** GO terms used to match gene ontologies from the high-confidence protein annotations reported in the new reference barley genome assembly published in 2017 (Mascher et al., 2017), i.e., GO_HV in the Venn diagram plot implemented in the VennDiagram R package (Chen and Boutros, 2011). The first source was AmiGO (Carbon et al., 2009). The second source was GO express (Rue-Albrecht et al., 2016).


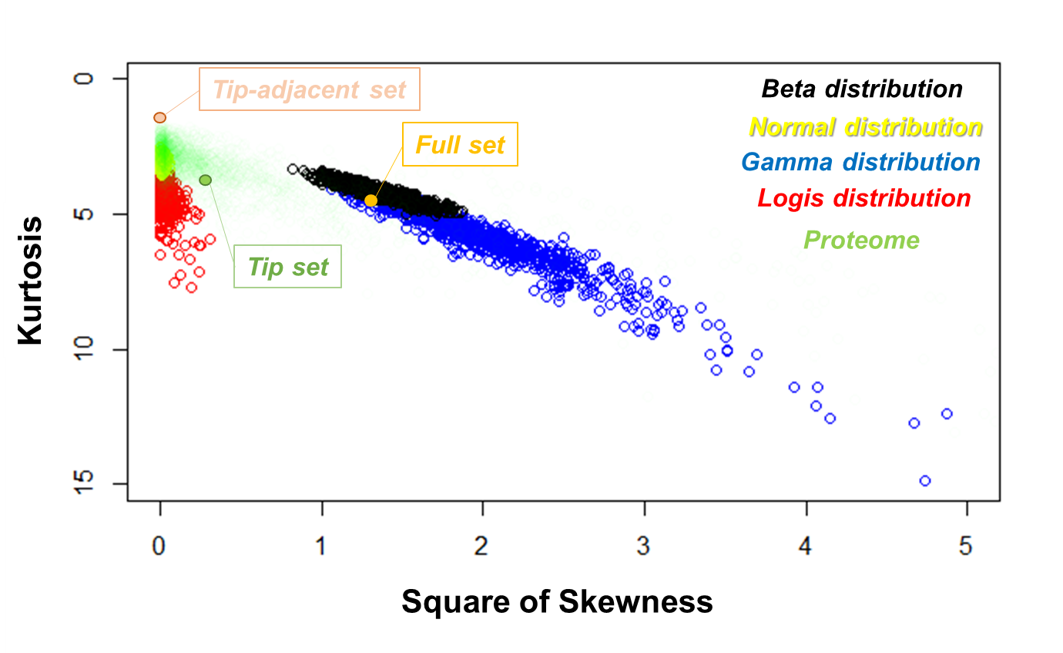


**Supplementary Figure 5.** **Distribution of protein-related response variables, namely protein content measured through the BCA assay and individual protein abundances measured through TMT-label assisted LC-MS/MS. Related to Figure 3; Figure 4; Supplementary Table 3; Supplementary Table 4.** The distribution of responses has been plotted along with exemplary distributions by calculating kurtosis and square of skewness, with functions from the R-GitHub repository RandodiStats (<https://github.com/MSeidelFed/RandodiStats>). Two root zones were measured separately, namely root tips and the upper tip-adjacent zone (**Figure 3** & **Table S3**). Note that both zones separately are near normally distributed. Whereas if we analyze both sets as one variable, the response features a gamma distribution. The green smear represents the distribution shapes of 2484 proteins (**Figure 4** & **Table S4**) slightly faded to enable visualization of other distributions. Note that many proteins follow a normal or near-normal distribution and as these get away from normality, the shape approximates a gamma distribution.

# Supplementary Tables

The tables have been deposited in separate .xlsx files due to their size, the legends and supplementary references are deposited in this document. The legends are also embedded in the .xlsx files.

**Supplementary Table 1. Master file containing all proteogenomics and proteomics *Hordeum vulgare* resources used during data analyses**. **Related to Supplementary Figure 4.** Despite barley being one of the first domesticated crops, the proteome coverage of manually curated entries in literature remains scarce as compared to other plant models. The UniProt database contains a variety of barley resources from both domesticated and wild *Hordeum vulgare* relatives. Only 0.175% of the entries correspond to Swiss-prot with varying degrees of experimental evidence supporting them (Total proteins: TrEMBL 210,767; Swiss-prot 368). We used the Swiss-prot proteins as the core of our FASTA dataset by aligning the amino acid sequences to the high-confidence annotations from the barley proteogenome (<http://pgsb.helmholtz-muenchen.de/plant/barley/index.jsp>) (Mascher et al., 2017) (**Gene sequences S1A - TAB**). Several UniProt entries mapped to the same entry and hence we calculated a matching score per alignment to judge how similar the sequences are (Durbin et al., 1998; Malde, 2008). The published data contains 39734 proteins and 1700 gene ontology (GO) terms with their specific definitions. GO terms were obtained by intersecting GOexpress (Rue-Albrecht et al., 2016) and AmiGO ontologies (Carbon et al., 2009) (**GO terms S1B - TAB**).

**Supplementary Table 2. Cellular component GO enrichment analysis comparing protein extraction methods in barley roots. Related to Figure 2; Supplementary Figure 2.** The Fisher exact test followed by the FDR correction was done using the gene ontology resource (Mi et al., 2019). S2A (**GO input TAB)** contains the *Hordeum vulgare* gene IDs from proteins with sequenced peptides during LC-MS/MS of root tips, common and unique proteins identified using two extraction methods, Lysis buffer (**LB**) with SDS and ribosome extraction buffer (**REB**) with mild detergents. S2B (**LB**), S2C (**REB**) and S2D (**Common**) TABS contain the outputs from the GO enrichment tests on the respective proteome fractions as analyzed through MaxQuant software (Cox and Mann, 2008). The GO terms are hierarchically sorted and can be interpreted as groups where the main category has been outlined in bold. The significant GO terms were then inputted into REVIGO (Supek et al., 2011) in order to remove redundant terms and visualize the results in semantic similarity-based scatterplots, which are presented in each tab.

**Supplementary Table 3. Bicinchoninic acid (BCA) assay results of tests conducted on the purified Hordeum vulgare root proteome. Related to Figure 3; Figure 4; Supplementary Figure 5**. BCA was done right after protein precipitation, cleaning and resuspension (**S3A** & **B**) and of tryptic peptides after reduction, alkylation and trypsin digestion (**S3C**). Ribosomal protein content was measured after ribosome enrichment and protein-rRNA dissociation using a chaotropic agent (**S3D**). Absorbance was measured in a plate reader at 562nm wavelength. The protein abundances were derived from blank corrected, dilution corrected, linear regression fitted values. The linear regression was performed using bovine serum albumin (BSA) as standard. Finally, the protein abundances were transformed into µg/mg fresh weight after dividing the values by their initial weight (W_0_) (**S3E**).

**Supplementary Table 4. Normalized protein intensities and statistics. Related to Figure 4; Supplementary Figure 3**. The normalized Log_2_ intensities are sorted according to a bootstrapped HCA (n = 10,000). Clusters of samples that are highly supported by the data (*AU - P* values > 95%) have been designated by red and black transitions of font colors (4 clusters, columns B-P; Q-AE; AF-AJ; AK-AN). Subsequently the same clustering method was used to find supported clusters of proteins, column AP contains feature IDs, which equal the edge number at each branch partition, of the mapped clusters above an *AU - P* value of 99%, the adjacent column has a compiled K-means clustering partition using five centers as an interpretation aid for the highly supported clusters. For class comparison a GLM was fitted taking each protein as a response variable and evaluating the necessary assumptions (column BG) of homoscedasticity and normality. Subsequently the appropriate test was applied, *P* values obtained (columns AQ-AX) and corrected with the FDR - BH 95´ (columns AY-BF).

**Supplementary Table 5. GO terms associated with statistically generated clusters. Related to Figure 5.** Clusters were produced by K-means (X1, X2, X3) or by bootstrapping a hierarchical cluster analysis and grabbing only those groups that appear more than 99% of the times (X109, X1347, X151, X1632, X614, X733, X904). Only groups of at least five proteins were inputted into a gene ontology enrichment analysis (Mi et al., 2019) followed by a semantic summary in REVIGO (Supek et al., 2011). Cluster GOs are divided by color transitions, each color belongs to a cluster indicated in column L "Cluster ID".

**Supplementary Table 6. Protein inventory within statistically relevant GO functional groups. See also Figure 5 and Table 1.** Statistical filters were implemented in a log2-transformed, pooled- and TMT-normalized matrix in order to select proteins with similar relative changes. The former procedure generated clusters of HORVU identifiers with similar responses that were then used as input for a GO enrichment test. Then functional groups were used to infer a group mean response and its respective quantiles here outlined as boxplots across the eight experimental conditions. Boxplots outline the group standard deviation across replicates, complementing the relative abundances outlined as a heatmap in Table 1.

**Supplementary Table 7A. General status of translation-related proteins in barley roots during experimental treatments of sustained cold stratification / acclimation and mimic biotic stress. Related to Figure 6; Table 2.** Barley protein matches were aligned to the whole Arabidopsis reviewed proteome (SwissProt - UniProt) and the maximum match score was used as the putative identity of the protein after corroborating that the FASTA ID for both Barley and Arabidopsis matched. The alignment was done using the pairwiseAlignment function of the package Biostrings in R. A generalized function to align two FASTA files has been made available in a GitHub repository (<https://github.com/MSeidelFed/Align2FASTAs>). Duplicated entries are explained in the **S7B TAB**.

**Supplementary Table 7B.** Matches with a score below ten were discarded in a first filtering step. In red non-unique or unique peptides that matched to more than one or to the same protein respectively. Note that all the red highlights correspond to isoforms or paralogs within the same RP or RAP family. Green font indicates proteins that have been manually mapped based on the Arabidopsis match since these did not appear in the BG2018 Master File (Beine Golovchuk et al., 2018).

**Supplementary Table 7C.** RP families and paralogs identified in this study. Paralogs with unique peptides are separated by "and", paralogs with non-unique peptides are separated by "or".

# Supplementary References

Beine Golovchuk, O., Firmino, A. A. P., Dąbrowska, A., Schmidt, S., Erban, A., Walther, D., et al. (2018). Plant temperature acclimation and growth rely on cytosolic ribosome biogenesis factor homologs. *Plant Physiol.* 176, 2251–2276. doi:10.1104/pp.17.01448.

Carbon, S., Ireland, A., Mungall, C. J., Shu, S., Marshall, B., Lewis, S., et al. (2009). AmiGO: Online access to ontology and annotation data. *Bioinformatics* 25, 288–289. doi:10.1093/bioinformatics/btn615.

Chen, H., and Boutros, P. C. (2011). VennDiagram: A package for the generation of highly-customizable Venn and Euler diagrams in R. *BMC Bioinformatics* 12, 1–7. doi:10.1186/1471-2105-12-35.

Cox, J., and Mann, M. (2008). MaxQuant enables high peptide identification rates, individualized p.p.b.-range mass accuracies and proteome-wide protein quantification. *Nat. Biotechnol.* 26, 1367–1372. doi:10.1038/nbt.1511.

Durbin, R., Eddy, S. R., Krogh, A., and Mitchison, G. (1998). *Biological Sequence Analysis*. doi:10.1017/cbo9780511790492.

Gu, Z., Eils, R., and Schlesner, M. (2016). Complex heatmaps reveal patterns and correlations in multidimensional genomic data. *Bioinformatics* 32, 2847–2849. doi:10.1093/bioinformatics/btw313.

Malde, K. (2008). The effect of sequence quality on sequence alignment. *Bioinformatics* 24, 897–900. doi:10.1093/bioinformatics/btn052.

Mascher, M., Gundlach, H., Himmelbach, A., Beier, S., Twardziok, S. O., Wicker, T., et al. (2017). A chromosome conformation capture ordered sequence of the barley genome. *Nature* 544, 427–433. doi:10.1038/nature22043.

Mi, H., Muruganujan, A., Ebert, D., Huang, X., and Thomas, P. D. (2019). PANTHER version 14: More genomes, a new PANTHER GO-slim and improvements in enrichment analysis tools. *Nucleic Acids Res.* 47, D419–D426. doi:10.1093/nar/gky1038.

Rue-Albrecht, K., McGettigan, P. A., Hernández, B., Nalpas, N. C., Magee, D. A., Parnell, A. C., et al. (2016). GOexpress: An R/Bioconductor package for the identification and visualisation of robust gene ontology signatures through supervised learning of gene expression data. *BMC Bioinformatics* 17, 126. doi:10.1186/s12859-016-0971-3.

Supek, F., Bošnjak, M., Škunca, N., and Šmuc, T. (2011). Revigo summarizes and visualizes long lists of gene ontology terms. *PLoS One* 6, e21800. doi:10.1371/journal.pone.0021800.
